# Knowledge gaps in tubular gut tumours: a critical appraisal of the 6th edition of the World Health Organization classification of tumours

**DOI:** 10.1002/path.70089

**Published:** 2026-07-07

**Authors:** Iris D Nagtegaal, Elizabeth A Montgomery, Alexandros D Polydorides, Anthony J Gill, Motohiro Kojima, Mark J Arends

**Affiliations:** ^1^ Department of Pathology Radboudumc Nijmegen The Netherlands; ^2^ Department of Pathology University of Miami Miller School of Medicine Miami FL USA; ^3^ Department of Pathology, Molecular, and Cell‐Based Medicine Icahn School of Medicine at Mount Sinai New York NY USA; ^4^ NSW Health Pathology, Department of Anatomical Pathology Royal North Shore Hospital St Leonards NSW Australia; ^5^ Sydney Medical School University of Sydney Sydney NSW Australia; ^6^ Department of Surgical Pathology Kyoto Prefectural University of Medicine Graduate School of Medical Science Kyoto Japan; ^7^ Edinburgh Pathology, Institute of Genetics & Cancer University of Edinburgh Edinburgh UK

**Keywords:** gastrointestinal, tumours, cancers, precursors, knowledge gaps, research questions

## Abstract

Gastrointestinal cancer is a global health problem. In the new 6th edition of the World Health Organization Classification of Tumours (WCT) of the Digestive System, updated evidence and guidance is provided for the aetiology, pathogenesis, diagnosis, classification, grading, staging and prognosis of these tumours. However, significant knowledge gaps remain, some of which are addressed in this review. To provide for a research framework to fill these knowledge gaps, we focused on precursor lesions and rare cancers, where the need for new evidence is most urgent. Areas with a need for further research are discussed for neoplastic precursors of the oesophagus (squamous epithelium), stomach (gastric dysplasia and adenomas), ampulla‐duodenum, jejunoileum, anal canal, as well as for colorectal serrated polyposis and inflammatory bowel disease associated dysplasia (conventional and non‐conventional). Knowledge gaps are also presented as they exist in rare cancers of the oesophagus (mixed type), appendix (mucinous neoplasms and mucinous adenomas), colorectum (three new subtypes) and anus, as well as for neuroendocrine tumours (grading, necrosis and two new gastric subtypes) and undifferentiated carcinomas (deficiencies of the SWI/SNF chromatin remodelling complex and mesenchymal differentiation). We identify and present research questions and persistent challenges for these entities and thus propose research frameworks that focus on specific priority areas. Progress in these core research topics for both neoplastic precursors and rare cancers will hopefully be reflected in future editions of the WCT, but many of these knowledge gaps will only be resolved with collective registries and collaborative research efforts. © 2026 The Author(s). *The Journal of Pathology* published by John Wiley & Sons Ltd on behalf of The Pathological Society of Great Britain and Ireland.

## Introduction

Gastrointestinal cancer is an increasing major health and economic burden, with nearly 3.5 million global cases annually, which is approximately 20% of the overall cancer burden and resulted in over 2 million deaths globally in 2022 [1]. To limit the impact of these types of cancer, adequate and accurate diagnoses are essential for optimal treatment. Insights into aetiology and pathogenesis may lead to preventative actions, risk reduction and early detection, as evident from colorectal cancer screening programmes [2, 3, 4]. Improved knowledge of gastrointestinal neoplasms at the population level is essential for health care planning and guideline development. Increasing insights into and understanding of the molecular pathological changes, both somatically acquired and inherited (germline/constitutional), that drive or are associated with progression of these neoplasms, are important for the development of new therapies as well as novel detection and monitoring methods, such as use of circulating cell‐free DNA or similar technologies.

The advent of a novel (6th) edition of the World Health Organization Classification of Tumours (WCT) of the digestive system [5] brings us one step further on the way towards optimal diagnosis of cancers. A critical appraisal of the available evidence in the 6th edition of the WCT of the digestive system reveals numerous knowledge gaps that stimulate research questions. In all chapters of the book, for all entities, these knowledge gaps and research questions can be identified. The level of knowledge in the common cancers (oesophageal adenocarcinomas and squamous cell carcinomas, gastric adenocarcinomas, colorectal adenocarcinomas) is higher than for both precursor lesions and relatively rare cancers. For the common cancers, routine reporting is well established and there are datasets from the International Collaboration for Cancer Reporting (ICCR) available [6, 7, 8, 9, 10, 11] that guide collection of the evidence for gross pathological and histopathological features that facilitate standardisation of diagnosis and reporting of pathological grading, staging, prognostic and other relevant factors. Therefore, in this review we focus on precursor lesions and rare cancers of the tubular gastrointestinal tract as described in the 6th edition of the WCT of the digestive system. Our aim is to discuss the current state of the field and to identify knowledge gaps, unique challenges and propose key priority research areas and questions.

## An overview of neoplastic precursors – current state of the field

### Epidermoid metaplasia of the oesophagus

The main precursor of oesophageal adenocarcinoma is Barrett's oesophagus. In recent years, remarkable progress has been made in understanding the stepwise progression of this condition. Histologically confirmed Barrett's oesophagus is present in approximately 1–2% of the general population [12, 13], but is considerably higher in high‐risk groups. Progression to oesophageal adenocarcinoma is low, approximately 0.3% per year [14]. Diagnosis is standardised with institution of expert panels and double reading [15, 16]. Surveillance and treatment is evidence based [17] and population‐based screening programmes are discussed [18, 19, 20].

In contrast, little is known about the precursors for oesophageal squamous cell carcinoma. In the 6th edition of the WCT, epidermoid metaplasia has been introduced as a probable precursor to squamous cell carcinoma; it is associated with squamous neoplasia [21, 22, 23] but the risk of progression remains unknown (Table 1). The same risk factors as those for squamous cell carcinoma have been documented, namely tobacco smoking, alcohol consumption and lichen planus, and it is sometimes detected in the course of evaluation of Barrett's oesophagus [21, 22, 23, 24, 25, 26]. Microscopically, it is characterised by sharply demarcated zones that display hyperkeratosis, mild acanthosis and a granular cell layer mimicking skin but without associated skin appendages. *TP53* mutations have been detected in epidermoid metaplasia and synchronous and metachronous squamous neoplasia [26]. Surveillance recommendations [27] include surveillance at 6 months and annually thereafter if no dysplasia is identified.

**Table 1:** Overview of definitions, aetiology and currently used biomarkers of the discussed precursor lesions.

Location	Entity	Definition	Aetiology/risk factors	Essential features and biomarkers
Oesophagus	Epidermoid metaplasia	A sharply demarcated area of epithelial hyperplasia with a prominent granular cell layer and hyperorthokeratosis	Risk factors: smoking, reflux, alcohol, lichen planus	Morphology
Stomach	Gastric dysplasia– intestinal type	An unequivocal neoplastic lesion of the gastric epithelium with intestinal metaplasia without stromal invasion	Risk factor: *Helicobacter* infection, hereditary	Primarily morphology based, but MUC2+. CD10+, cdx2+
	Gastric dysplasia – foveolar type	An unequivocal neoplastic lesion of the gastric epithelium without stromal invasion	Unclear, part hereditary	Primarily morphology based, but MUC5AC+, MUC6+
	Pyloric gland adenoma	A non‐invasive gastric epithelial neoplasm consisting of dysplastic glands with pyloric/mucous neck cell‐type differentiation	Risk factors: autoimmune and *Helicobacter‐*associated gastritis, hereditary	Morphology, variable expression of immunohistochemical markers
	Oxyntic gland adenoma	Intramucosal neoplasm composed of cells with mild cytologic atypia, predominantly differentiating into chief cells, along with a variable proportion of cells exhibiting differentiation toward parietal cells and mucous neck cells	Unclear	Morphology, MUC6+, Cyclin D1+
Colon	IBD‐associated dysplasia	Neoplastic alteration of the intestinal epithelium confined within the basement membrane and arising in colorectal mucosa damaged by long‐term IBD	Risk factors related to IBD	Various subtypes, role for p53 immunohistochemistry and aneuploidy detection
Anus	Anal squamous intraepithelial neoplasia	HPV‐associated non‐invasive neoplasms of the anal canal and the perianal region	Risk factor: HPV infection (HPV independent AIN occurs)	P16 immunohistochemistry

AIN, anal intraepithelial neoplasia; HPV, human papillomavirus; IBD, inflammatory bowel disease.

### Gastric dysplasia and adenomas

In the new edition of the WCT, sections previously dedicated to gastric dysplasia, intestinal type adenomas and gastric type adenomas are combined into a single section *gastric dysplasia*, defined as unequivocal neoplastic lesions of the gastric epithelium without stromal invasion, following the MAPS III guidelines (Management of epithelial precancerous conditions and lesions in the stomach) (Table 1) [28]. Previously, raised dysplastic foci with intestinal differentiation had been classified as adenomas. Now, it has been recognised that such lesions can be considered analogous to polypoid colitis‐associated colorectal dysplasia. They arise in damaged mucosa, in contrast to colorectal adenomas, which typically arise in normal mucosa. It is therefore no longer deemed appropriate to call these lesions adenomas. Gastric dysplasia with intestinal differentiation, whether flat or polypoid (formerly intestinal type adenoma), is generally detected in patients with background mucosal atrophy and intestinal metaplasia, usually in the incisura and antrum. A minority (about 10%) arise in the body and fundus. Occasionally foveolar‐type dysplasia also arises in *Helicobacter pylori*‐associated gastritis [29, 30, 31, 32].

Foveolar‐type dysplasia generally arises in *H. pylori*‐naïve patients (individuals who have never had *H. pylori* infection) on the surface of oxyntic mucosa of the gastric body/fundus. The same anatomical predilection mirrors that of foveolar‐type dysplasia arising from polyps in patients with familial adenomatous polyposis (FAP) and the gastric adenocarcinoma and proximal polyposis of the stomach variant thereof, due to pathogenic variants in the *APC* promoter [29, 33, 34].

### Gastric oxyntic and pyloric gland neoplasms

Two rare forms of gastric dysplasia that predominantly arise deep to the surface epithelium are gastric pyloric gland adenoma (PGA) and oxyntic gland adenoma, and these are described separately in the WCT (Table 1) [5].

Sporadic gastric PGAs classically, but not invariably, arise in the setting of pyloric gland metaplasia in oxyntic mucosa with atrophic gastritis, often of the autoimmune type [35, 36, 37]. Syndromic PGAs associated with FAP are not usually associated with a metaplastic background. Both sporadic and FAP‐associated PGAs consistently feature *GNAS*, *KRAS* and *APC* mutations [38, 39, 40, 41]. They consist of closely packed tubules or glands containing bubbly cytoplasm that imparts a ground glass appearance with round nuclei. Progression to high‐grade dysplasia/adenocarcinoma is more frequent in elderly patients with autoimmune gastritis. The neoplastic risk increases with size, tubulovillous architecture, and a mixed immunophenotype. Prognosis depends on any carcinomatous component; however, after complete endoscopic or surgical resection, the overall local recurrence rate is < 10% [42].

Oxyntic gland neoplasms [43] include a spectrum of adenomas (restricted to the mucosa with minimal cytologic atypia) and carcinomas that have been termed gastric adenocarcinoma of fundic type (extending into submucosa with increased cytologic alterations). They are rare, featuring tightly packed angulated glands with chief cell differentiation, parietal cell differentiation or both. The chief cell predominant pattern is the most common, composed of a glandular proliferation of monotonous columnar cells with basophilic cytoplasm. On immunohistochemical staining, they express MUC6, but not MUC5, in contrast to PGA, which co‐expresses the two. Cyclin D1 is expressed, which can be exploited for diagnosis [44]. Their aetiology remains unclear, but they are considered *H. pylori* independent [45] and appear to carry favourable prognosis even when progressing to gastric adenocarcinoma of fundic gland type [43, 45].

### Small bowel precursors

Small intestinal neoplasms most commonly occur in the duodenum, particularly around the ampulla and descending duodenum [46, 47, 48, 49]. Ampullary precursor lesions, termed ampullary adenomatous neoplasms, may evolve into invasive adenocarcinomas that are subclassified primarily by anatomical site into four categories, although some have additional macroscopic and histopathological features: (1) non‐ampullary adenocarcinoma, (2) ampullary‐duodenal adenocarcinoma, commonly presenting as an exophytic mass growing on the duodenal surface of the ampulla obstructing the orifice of the papilla, (3) intra‐ampullary papillary‐tubular neoplasm‐associated adenocarcinoma, with a typical intra‐ampullary growth of a papillary precursor adenoma adjacent to an adenocarcinoma, and (4) ampullary ductal adenocarcinoma that mostly originates from flat, high‐grade intraepithelial neoplasia of the ampullary ducts [50, 51, 52, 53, 54]. Non‐ampullary adenocarcinomas and ampullary‐duodenal adenocarcinomas are often associated with an adenomatous component [55].

Adenomas in the jejunum and the ileum share morphological features with colorectal counterparts, although there is considerably less evidence about these much less frequent small intestinal neoplasms. Poor accessibility for endoscopic biopsy and resection has resulted in few samples available for research, indicating a significant knowledge gap that may require multicentre studies to collect a sufficient number of cases for research investigation.

#### Serrated polyposis

Serrated polyposis is a condition of unknown aetiology characterised by multiple serrated polyps in the colon and rectum and associated with an increased risk of colorectal carcinoma [5]. Over the last two decades or so, it has become increasingly clear that some rare instances of colorectal serrated polyposis may be caused by inherited germline (constitutional) pathogenic variants (or rare *de novo* variants) in a single confirmed gene, *RNF43*, accounting for around 2% of patients [56, 57, 58]. Despite familial case collections from various research groups, no other high‐penetrance candidate genes have been identified so far. For example, no germline mutations were detected in any of the well‐known polyposis genes in a recent study of 65 patients with serrated polyposis [59]. Large‐scale genetic studies have also failed to identify high‐penetrance candidate genes [60, 61]. Therefore, serrated polyposis is now viewed as a largely sporadic syndrome and thus no longer included in the WCT chapter of genetic tumour syndromes of the digestive system.

However, serrated polyposis or multiple serrated polyps may be present as components of other polyposis syndromes, including *PTEN* hamartomatous tumour syndrome and hereditary mixed polyposis syndrome [62, 63, 64]. Whether there are other rare or very rare inherited genetic causes of serrated polyposis remains a question. More significantly, there are knowledge gaps concerning environmental and lifestyle factors, including cigarette smoking and high body mass index [65], that may contribute to sporadic serrated polyposis, but the exact aetiology is unknown. Similarly, there is little known about the risk of progression of such serrated polyps to malignancy, even though in patients with serrated polyposis and colorectal carcinoma around half have a *BRAF* mutation, 40% are MLH1 deficient and < 5% have a *KRAS* mutation [66, 67]. Finally, it is not clear what would be the most effective and efficient surveillance strategy for serrated polyposis.

#### Inflammatory bowel disease: conventional and non‐conventional dysplasia

**Figure 1:**
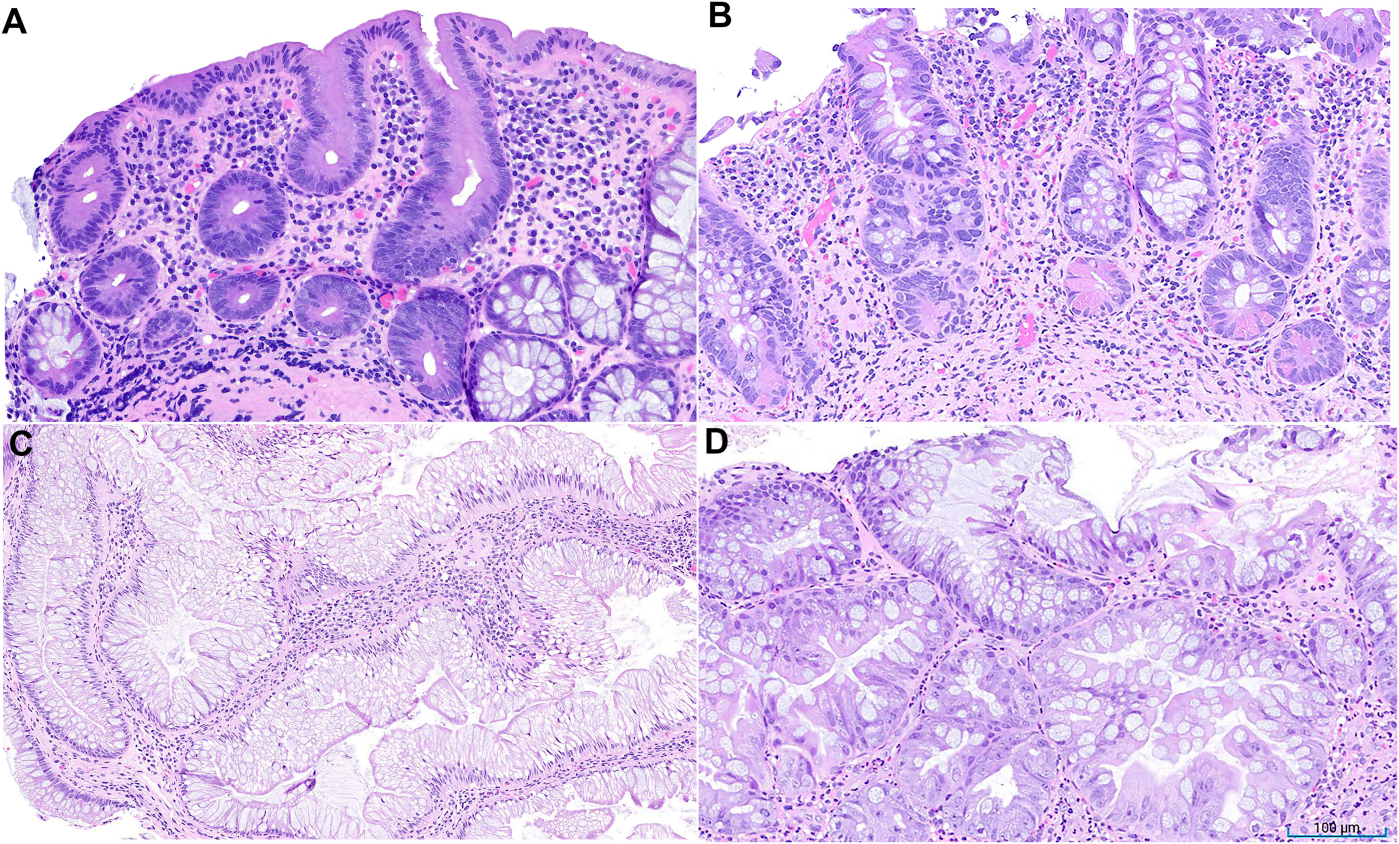
Knowledge gaps in inflammatory bowel disease related dysplasia relate mainly to non‐conventional (non‐adenomatous, non‐intestinal) dysplasia. (A–D) Microscopic illustrations of the main patterns of non‐conventional dysplasia. (A) Goblet‐cell deficient dysplasia is characterised by distinct, sharply demarcated dysplastic epithelium with mucin‐depleted eosinophilic cytoplasm and almost complete lack of goblet cells. (B) Crypt cell dysplasia (also called ‘with terminal differentiation’) is composed of dysplastic cells with polymorphic phenotypes that recapitulate the appearance of colonic crypts, including Paneth cells, endocrine cells and goblet cells (which are often ‘dystrophic’ or ‘upside‐down’). (C) Hypermucinous dysplasia comprises long villous projections of exuberant epithelium with abundant mucin, monotonous nuclei with low‐grade atypia (that often mature further towards the surface) and cytoplasm that is often gastric/foveolar in phenotype (this pattern is also called gastric‐type dysplasia). (D) Serrated dysplasia encompasses all phenotypes with a serrated appearance, including lesions that resemble sporadic traditional serrated adenoma (TSA‐like), dysplasias that are similar to sessile serrated lesions with dysplasia (SSLD‐like), as well as lesions (such as the one shown here) that do not belong in a specific category, but clearly demonstrate dysplastic epithelium with serrated architecture (i.e., serrated, not otherwise specified—NOS). (Scale bar, 100 μm).

Patients with inflammatory bowel disease (IBD), including ulcerative colitis (UC) and Crohn's disease (CD), have an increased risk of colorectal neoplasia. Although lower than originally thought, the increased risk is mostly attributable to overall cumulative inflammatory burden. Therefore, it is higher in patients with earlier disease onset, longer disease duration, more extensive anatomical involvement of the colon and more severe inflammation [68]. Dysplasia is both a precursor (Figure 1, Tables 1 and 2) and a risk factor in the development of invasive carcinoma, so endoscopic surveillance in patients with IBD is centred on its identification and management, with histopathological diagnosis a key component [69]. Nevertheless, there is considerable interobserver variability among pathologists for both diagnosis and grading [70, 71]. Morphologically, most dysplasia in patients with IBD resembles sporadic conventional colorectal adenomas with tubular and/or villous architecture. Although it might differ from sporadic adenomas in its molecular signature, this pattern is referred to as ‘conventional’, ‘intestinal’ or ‘adenomatous’ dysplasia and graded according to the Vienna classification of all gastrointestinal neoplasia or a similar scheme which was specifically designed for IBD lesions [72, 73]. For low‐grade dysplasia (and in some instances even high‐grade dysplasia) of this type, management largely rests on the extent of the endoscopists' ability to recognise and remove it [69].

**Table 2:** Overview of differences between conventional (adenomatous) dysplasia and non‐conventional dysplasia in IBD.

Characteristic	Adenomatous dysplasia	Non‐adenomatous dysplasia
Epidemiology/risk factors	Longer disease duration/earlier onset, anatomic extent and severity of inflammation, concomitant PSC	Some studies suggest higher percentage in paediatric patients and adults with PSC, but not confirmed
Endoscopy/macroscopy	Visible/invisible, polypoid/nonpolypoid, resectable/unresectable (Paris/SCENIC classification)	Some studies suggest higher percentage of flat or invisible lesions, but not confirmed
Histology – subtypes	Tubular, tubulovillous, villous	Hypermucinous/gastric, serrated, goblet cell deficient, crypt cell type/terminally differentiated
Histology – grading	Low grade or high grade (Vienna or Riddell systems)	No validated or established grading criteria exist
Molecular/IHC	TP53 is early alteration, IHC may help identify p53‐mutant lesions which is not, however, entirely sensitive or specific for dysplasia (field effect/cancerisation)	Some studies suggest higher percentage of molecular abnormalities (e.g., aneuploidy, KRAS mutations, etc.), but not confirmed
Prognosis/outcomes	Depends on dysplasia grade and endoscopic appearance and resectability (visible and polypoid lesions fare better)	Some studies suggest more aggressive behavior, but not confirmed

IBD, inflammatory bowel disease; IHC, immunohistochemistry; PSC, primary sclerosing cholangitis.

There are several additional morphological patterns of IBD‐related non‐conventional dysplasia that have been more recently recognized. These new patterns may be more difficult to recognise, diagnose and grade [74, 75, 76]. Originally and collectively referred to as non‐conventional dysplasia, these patterns are better separated based on morphology, for several reasons: (1) likely better recognition by pathologists when specifically defined; (2) more appropriate subtyping in order to study their possibly independent clinico‐pathological features and molecular pathological underpinnings; (3) more suitable clinical and endoscopic management. Although a few different definitions and classification systems exist and mixed patterns are very common, non‐adenomatous dysplasia in IBD usually falls into one of four distinct categories: (a) goblet cell‐deficient dysplasia (also called eosinophilic), (b) hypermucinous dysplasia (also called gastric, particularly when accompanied by foveolar phenotype), (c) serrated dysplasia (including sessile serrated lesion‐like, traditional serrated adenoma‐like, and serrated not otherwise specified), and (d) crypt cell dysplasia (also called terminally differentiated and including a Paneth cell‐rich variant).

Knowledge gaps associated with these non‐adenomatous types of dysplasia in patients with IBD arise in a few areas. The diagnosis itself needs refinement, particularly in terms of defining minimum criteria coupled with inter‐observer reproducibility studies [74]. Such studies will facilitate better endoscopic recognition, molecular characterisation and prediction of their natural history; and therefore, improve clinical management. As with other lesions in IBD, the most important confounding factor is the apparent field effect whereby large areas of colonic mucosa show clonal molecular changes in the epithelium that might be able to expand and progress, such as those due to mutated *TP53* or *KRAS* [77, 78, 79]. This limits the ability to study the significance and contribution of individual lesions to neoplastic risk. For example, any apparent increased risk of progression may actually be attributable to coexistent field effect changes or other flat lesions elsewhere which may not have been recognised endoscopically [80, 81, 82].

#### Anal intraepithelial neoplasia

**Figure 2:**
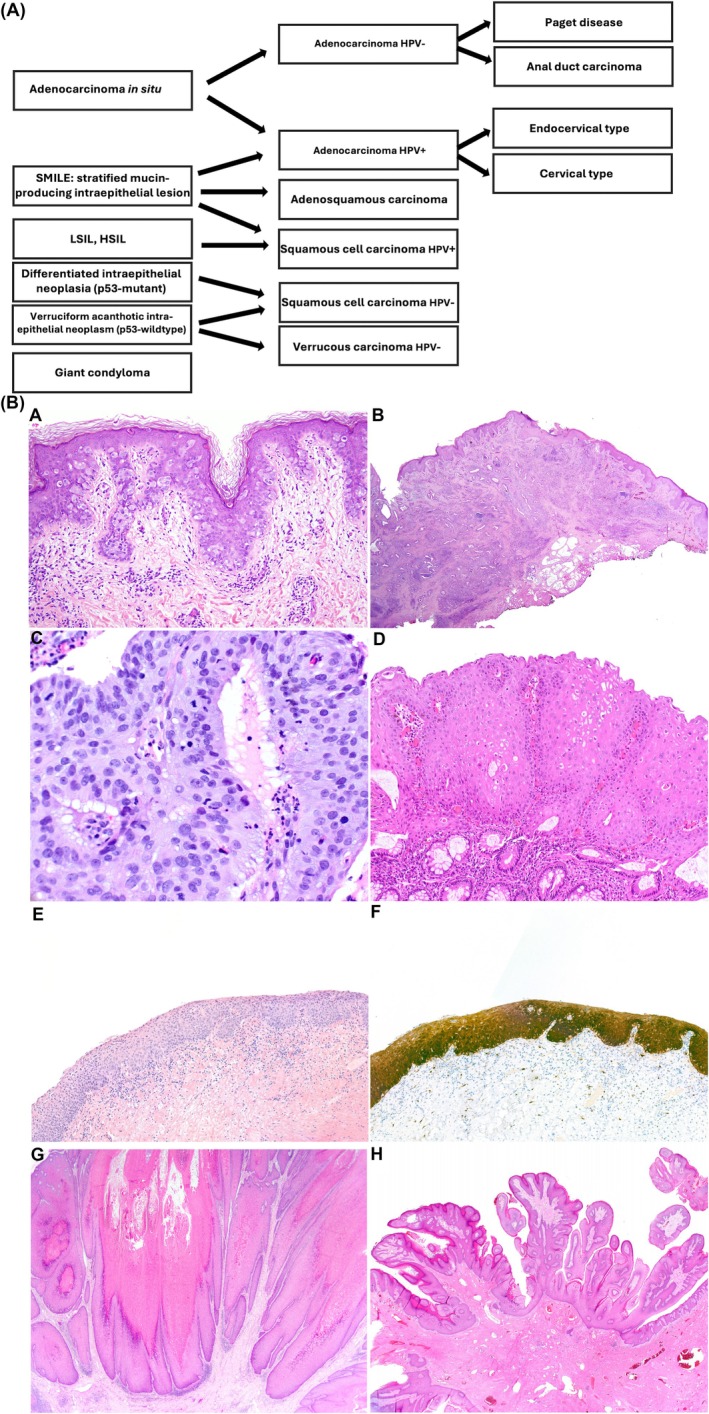
Anal precursors and cancers. (A) Schematic overview of the histological progression models for the different types and subtypes of anal precursor and cancer. (B) Histological example images of anal precursors and cancers (image files in brackets): (A) anal Paget disease, (B) anal duct carcinoma, (C) anal SMILE, (D) anal low‐grade squamous intraepithelial lesion (LSIL), (E) anal high‐grade squamous intraepithelial lesion (HSIL), (F) anal HSIL P16 immunostain, (G) anal verrucous carcinoma, (H) anal giant condyloma.

The majority of anal intraepithelial neoplasia (AIN; Figure 2 and Table 1) is caused by human papillomavirus (HPV) infection, for which the Lower Anogenital Squamous Terminology (LAST) system is incorporated [83]. Low‐grade squamous intraepithelial lesion (LSIL) includes lesions previously classified as mild dysplasia, anal intraepithelial neoplasia/IEN I (AIN I), anal squamous intraepithelial lesion I and condyloma acuminatum. LSIL shows cytological atypia and mitotic figures in the lower third of the epithelium only, and koilocytotic atypia. Condyloma acuminatum, a subset of LSIL, is exophytic with acanthosis, broad rete pegs, parakeratosis and koilocytotic atypia. LSIL and high‐grade squamous intraepithelial lesion (HSIL) may be components of anal condylomas due to infection with multiple HPV subtypes [84, 85, 86]. HSIL encompasses moderate dysplasia, severe dysplasia, carcinoma *in situ*, Bowen disease and Bowenoid papulosis. It shows involvement of two thirds or more of the squamous epithelium by marked cytological atypia, mitotic figures in the upper two thirds of the epithelium, atypical mitotic figures and loss of nuclear polarity. p16 immunohistochemistry is typically diffusely ‘block’ positive [83]. For HPV‐independent preinvasive lesions there are considerable knowledge gaps [87].

### Knowledge gaps for gastrointestinal precursors: a research framework

**Figure 3:**
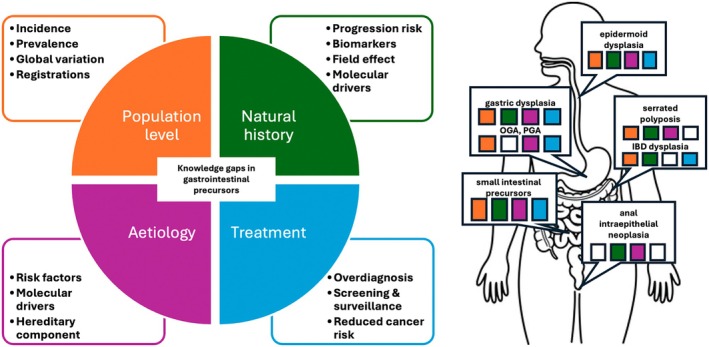
Research framework for gastrointestinal precursor lesions and knowledge gaps according to diagnosis. Knowledge gaps are indicated by the coloured boxes in the appropriate locations (white boxes represent insufficient knowledge) (diagram created with Microsoft PowerPoint). IBD, inflammatory bowel disease; OGA, oxyntic gland adenoma; PGA, pyloric gland adenoma.

When evaluating gastrointestinal precursor lesions, there is a contrast between those that are well studied (Barrett's oesophagus, colorectal conventional adenomas, serrated polyps) and those that are not. While for the former, clinical care is well organised with preventative actions being effectively implemented, the latter are significantly lagging behind. In Figure 3 we propose a research framework to reduce these knowledge gaps, which is essential for ultimately reducing the burden of gastrointestinal cancer.

In general, understanding of precursors at the population level is lacking. There is limited information about the incidence and prevalence of precursor lesions due to a lack of (inter)national registries. Where organised screening or surveillance programmes exist, we can estimate incidence in a crude way. For example, oesophageal epidermoid metaplasia is present in 0.18% of routine oesophageal biopsies [21] based on a single case series. For anal HSIL, prevalence in high‐risk groups is estimated to be between 7% and 55% [88], demonstrating considerable numerical variability. For these two examples, many risk factors are known and this might influence reported numbers. For other precursors, significantly less is known and, as a consequence, the global variation is less well understood.

The lack of epidemiological data hampers the determination of progression risk. Since precursors are generally considerably more numerous than their associated cancers, these data are necessary to determine the true progression risk. Progression risk is also dependent on other factors, including the presence and extent of a field effect, which is often directly related to the aetiology of the precursor. For hereditary tumour predisposition syndromes, the entire organ system is likely to be at risk, such as the whole colon and rectum in either Lynch syndrome, FAP or other polyposis syndromes, although particular risks may vary with position along the large intestine. Although individual lesions may not progress after complete resection, the development and subsequent progression of other metachronous precursor lesions warrants continued surveillance. If the key aetiological risk factor is related to chronic inflammation, as in gastric dysplasia [28] and IBD‐related dysplasia [89], or an infectious agent, as in anal neoplasia [81], either part or the entire organ may be at risk and thus appropriate surveillance, preventative measures and/or therapies may be necessary.

The study of the natural history of precursor lesions inevitably includes molecular investigations to identify drivers of progression and potential biomarkers. Currently, these are largely limited to immunohistochemistry for p53 [90, 91, 92] and p16 [93, 94], but more specific biomarkers that allow patient‐tailored risk stratification are in urgent clinical need. This would also prevent overdiagnosis, that is the detection of precursor lesions that do not indicate that a patient is at increased risk for developing an associated cancer, and thus treatment and/or surveillance of the patient may not be necessary. These ‘overdiagnosed’ lesions should therefore not be an indication for clinical action. Since this information is currently lacking for the majority of precursor lesions, due to knowledge gaps, the public health burden of these precursors of gastrointestinal cancers cannot be lessened by appropriate modifications to surveillance protocols.

### An overview of rare gastrointestinal cancers – current state of the field

The definition of rare cancers is highly variable and population dependent. The US National Institutes of Health National Cancer Institute defines rare cancers as cancers that occur in fewer than 15 out of 100,000 people each year [95], while the European Union states that cancers with an incidence of lower than 6 per 100,000 per year can be considered rare [96]. According to this latter definition, all primary cancers occurring in the oesophagus, small bowel, appendix and anus can be considered rare. In addition, uncommon cancer types that occur in common locations (i.e. rare subtypes of bowel cancer) can be considered rare cancers.

### Rare cancers in the oesophagus: mixed type

While by definition oesophageal cancer can be considered rare, for this review we restrict ourselves to the rare ‘mixed’ oesophageal carcinomas: mucoepidermoid carcinoma, adenoid cystic carcinoma and adenosquamous carcinoma (Table 3). In the 6th edition of the WCT of the digestive system, these three subtypes are grouped together. These lesions are rare, accounting for less than 1% of oesophageal carcinomas. Patients are usually men in their seventh decade of life or older [97, 98, 99, 100, 101, 102, 103]. Mucoepidermoid carcinoma and oesophageal adenoid cystic carcinoma are believed to arise in association with oesophageal submucosal glands [97, 104]. Adenosquamous carcinoma may arise from either a primary squamous cell carcinoma or primary adenocarcinoma, associated with Barrett's oesophagus. Neoplastic cells can develop glandular or squamous differentiation from a common precursor, and the two components share overlapping mutational profiles [105, 106]. Mucoepidermoid and adenoid cystic carcinomas are akin to salivary gland counterparts. Mucoepidermoid carcinoma shows cysts and sheets of mucin‐producing epithelial cells, epidermoid cells and intermediate cells in a range of quantities of each; *MAML2* gene rearrangements can be detected. Epithelial and myoepithelial cells in a bilayer with inner epithelial and outer myoepithelial cells organised in a variety of cribriform, tubular (glandular) or solid patterns characterise adenoid cystic carcinoma. A characteristic feature is the presence of crisply demarcated spaces filled with hyaline material. *MYB* gene rearrangements and overexpression of MYB protein on immunohistochemistry can be detected [107, 108]. Distinct adenocarcinoma and squamous cell carcinoma components that are separate or intermingled characterise adenosquamous carcinoma. An arbitrary requirement for at least 20% of either has been advocated (but probably lacks impact on outcome), otherwise, the majority component determines classification [109].

**Table 3:** Overview of rare cancers, including rare subtypes of the tubular gastrointestinal tract. Definitions, histopathology, aetiology, molecular background and outcome are summarised.

Location	Entity	Definition	Pathogenesis/precursor	Molecular background	Prognosis
Oesophagus	Mucoepidermoid carcinoma	A malignant tumour composed of a variable mixture of malignant mucous secreting cells, epidermoid cells and intermediate cells resembling its salivary gland counterpart and characterised by MAML2 gene rearrangements	Neoplastic derivation from submucosal glands	*MAML2* gene fusion arrangements	Unknown
	Adenoid cystic carcinoma	A malignant tumour composed of epithelial and myoepithelial cells arranged in cribriform, tubular or solid architecture and punched out spaces filled with hyaline material	Neoplastic derivation from submucosal glands	*MYB* gene fusion arrangements	Unknown
	Adenosquamous carcinoma	A malignant tumour composed of distinct malignant squamous and malignant glandular components.	Divergent differentiation within an adenocarcinoma or a squamous cell carcinoma	Molecular features of oesophageal adenocarcinoma or a squamous cell carcinoma	Unknown
Appendix	LAMN and HAMN	Circumferential mucinous epithelial proliferation, with expansion without infiltrative growth	Unclear	*KRAS* and *GNAS* mutations co‐occurring in the majority of cases	Variable
Colorectal	Serrated adenocarcinoma	This subtype is defined by morphological similarities with serrated polyps, with glandular serration that can be accompanied by mucinous areas	From serrated precursors	CIMP, *BRAF* mutations	More data required
	Micropapillary carcinoma	This subtype is characterised by small clusters of inverted eosinophilic tumour cells within retracted stromal spaces mimicking vascular channels in at least 5% of the tumour	Unknown	Unknown	Poor outcome
	Medullary carcinoma	In this subtype >50% of the tumour is characterised by solid sheets of malignant cells with vesicular nuclei, prominent nucleoli, abundant eosinophilic cytoplasm and a high mitotic rate, exhibiting regions of necrosis and prominent infiltration by lymphocytes and neutrophils	Unknown	MSI	Good outcome
	Adenoma‐like adenocarcinoma	In this subtype >50% of the invasive areas demonstrate pushing invasion with villous adenoma‐like architecture and low‐grade cytologic features	Unknown	KRAS mutations	Good outcome
	Adenosquamous carcinoma	This tumour type shows histologic features of both adenocarcinoma and squamous cell carcinoma, in varying proportions	Unknown	MSI, TP53, APC mutations	Poor outcome
	Squamous cell carcinoma	A malignant epithelial tumour with squamous differentiation	Unknown	PTEN PIK3CA	Poor outcome
	Low‐grade tubuloglandular adenocarcinoma	This subtype infiltrates as haphazard, well‐differentiated glands without evidence of desmoplasia	IBD related	IDH1 mutations	Good prognosis
	Lymphoglandular complex‐like adenocarcinoma	This subtype is characterised by prominent associated lymphoid tissue	Unknown	MSI	Good prognosis
Anal	Squamous cell carcinoma	A malignant epithelial tumour originating from the squamous anal mucosa	HPV related (in 90% of cases)	PIK3CA mutations, loss of RB1 function (TP53 mutations in HPV independent cases)	Good–moderate prognosis
	Adenocarcinoma	An invasive neoplasm of the glandular epithelium of the anal canal	HPV related	KRAS	Poor outcome

HAMN, high‐grade appendiceal mucinous neoplasms; HPV, human papilloma virus; IBD, inflammatory bowel disease; LAMN, low‐grade appendiceal mucinous neoplasms.

### Appendiceal tumours

**Figure 4:**
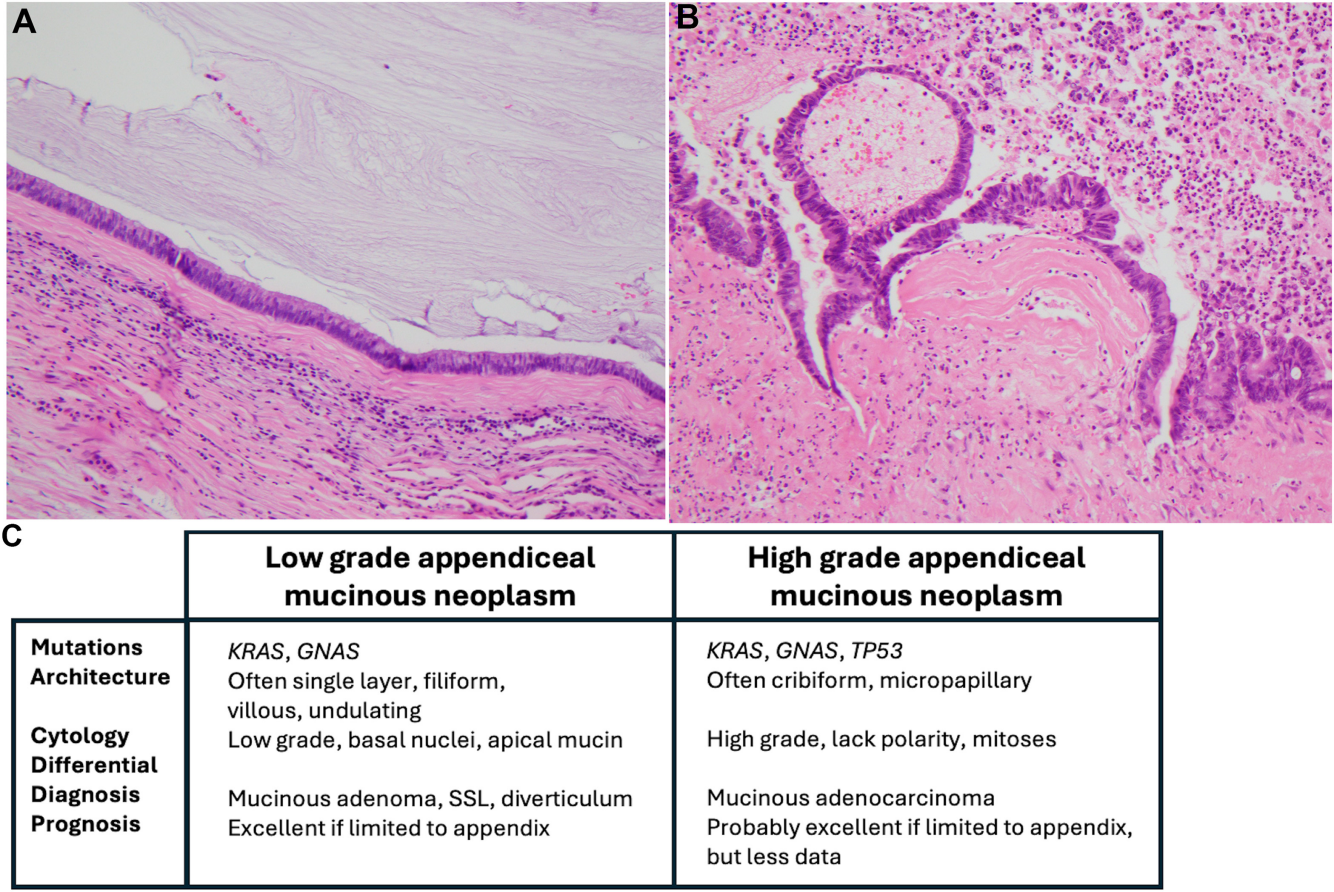
Overview of the differences between low‐grade appendiceal mucinous neoplasia (LAMN) and high‐grade appendiceal mucinous neoplasia (HAMN). (A) LAMN is lined by neoplastic cells that demonstrate only subtle nuclear atypia, equivalent to low‐grade dysplasia. (B) In contrast, HAMN is lined by cells demonstrating greater nuclear atypia, equivalent to high‐grade dysplasia. While HAMNs that progress to peritoneal involvement are more likely to be present with grade 2 mucinous carcinomatosis peritonei, there is little evidence to suggest that HAMNs have a greater risk of progression than LAMNs. (C) Comparison of the mutations, architecture, cytology, differential diagnosis and prognosis between LAMN and HAMN.

All appendiceal tumours are considered rare neoplasms. A research framework for all types has been recently described [110]. In the current review we focus on appendiceal mucinous neoplasms (AMN) (Figure 4 and Table 3). A change in the 6th edition of the WCT [5] concerns the re‐introduction of appendiceal mucinous adenomas, separately from AMN. The latter are subdivided into low‐grade appendiceal mucinous neoplasms (LAMN) and high‐grade appendiceal mucinous neoplasms (HAMN). This distinction of appendiceal mucinous adenoma from AMNs is intended to identify tumours which are reliably cured by appendicectomy alone, compared with those which might have a risk of synchronously or subsequently developing the clinical syndrome of pseudomyxoma peritonei (PMP), histopathologically known as mucinous carcinoma peritonei (MCP). Nevertheless, AMNs limited to the appendix demonstrate an excellent prognosis with little, if any, chance of recurrence [111, 112, 113, 114] and strong arguments can be made to combine AMNs limited to the appendix, at least low‐grade ones, with mucinous adenomas into a single stage with negligible metastatic risk [114].

The distinction between LAMN and HAMN historically rests on case reports of HAMN that subsequently behaved in an aggressive manner. However, it is likely that these cases represent *in situ* lesions associated with invasive adenocarcinoma in under‐sampled tumours [115]. There is now evidence that HAMN and LAMN share the same stage‐dependent outcome with an excellent prognosis after appendicectomy alone in cases confined to the organ's wall [114, 115, 116]. Therefore, there may be insufficient evidence to support their separation, other than to suggest that HAMNs that metastasize to the peritoneum are more likely to present with grade 2 MCP compared with LAMNs [115].

In the 6th edition [5], peritoneal metastases from appendiceal mucinous neoplasms and biologically similar entities (such as mucinous adenomas arising from mature ovarian teratomas) are separated from peritoneal metastases due to other malignancies. This distinction recognises that AMNs are the most common cause of the distinctive clinical syndrome of PMP, which is usually only treated in specialised centres using unique treatment approaches, such as hyperthermic intraperitoneal chemotherapy or cytoreductive surgery. However, whether such a separation is justified based on the natural history of metastatic mucinous adenocarcinomas of similar grades from different sites remains uncertain.

While there is a suggestion that molecular profiles can assist in prognostication – for example peritoneal metastasis by *RAS*‐mutated/*GNAS* wild‐type/*TP53* wild‐type tumours may have a more favourable outcome, particularly compared with *TP53*‐mutant tumours – this has been questioned by other studies [117, 118, 119, 120]. There is currently insufficient evidence to support molecular grading and classification of AMNs.

### Rare colorectal carcinomas

The 6th edition of the WCT of the digestive system [5] includes three new rare subtypes of colorectal cancer (Table 3). A new concept of intramucosal carcinoma [121, 122] has been introduced, similar to the intramucosal carcinomas of the upper gastrointestinal tract. Intramucosal adenocarcinoma is characterised by definite infiltration of tumour cells in colorectal mucosal lamina propria (and no deeper), with overtly poor differentiation, poorly differentiated clusters, signet‐ring carcinoma cells or tumour budding [121, 122]. Intramucosal carcinomas are indeed rare with a very low incidence (< 0.1% of all colorectal carcinomas), however there is a strong correlation with hereditary cancer predisposition syndromes [121].

Low‐grade tubulo‐glandular adenocarcinoma is a rare new morphological subtype with distinctive appearances, that usually arises in IBD from low‐grade conventional dysplasia or from non‐conventional colitis‐associated dysplasia in IBD [123]. Somatic *IDH1* mutations are common [124]. Rare colorectal cancers previously described as ‘dome carcinoma’ or ‘gut associated lymphoid tissue carcinoma’ in the older literature are currently defined in the 6th edition of WCT [5] of the digestive system as lymphoglandular complex‐like adenocarcinomas. They usually present at a low stage [125, 126] and are more frequently encountered in population screening programmes.

All of these newly defined tumour types, as well as serrated adenocarcinoma, micropapillary adenocarcinoma and adenoma‐like adenocarcinoma, present with knowledge gaps relating to accurate epidemiological data on incidence and prevalence, some risk factors concerning aetiology and progression risks during pathogenesis, including molecular pathology alterations in the precursors involved in progression to cancers. Answering these research questions will help to guide preventative measures, development or modification of surveillance programmes, early diagnosis and improved understanding of treatment options and prognosis.

### Anal carcinomas: squamous cell carcinoma and adenocarcinoma

All anal carcinomas are considered rare tumours (Figure 2 and Table 3), the majority of which are squamous cell carcinoma (SCC), with only 5–10% adenocarcinomas (mainly mucosal) [127, 128]. Most SCCs are HPV associated but some are HPV independent. Rising incidence rates are believed to reflect changing sexual behaviour and longstanding HPV infection in HIV‐positive patients [129, 130, 131], resulting in a sharp increase of 2.7% per year between 2001 and 2015. Women are more frequently affected, unrelated to HIV, whereas HIV is a key factor in men [132, 133, 134, 135]. Verrucous carcinoma (VC) is a rare subtype that is not associated with HPV infection. It shows acanthosis and a pushing interface with the underlying tissue. It lacks HPV cytopathic effect and shows minimal cytologic atypia. VC is not the same as giant condyloma of Buschke–Lowenstein, which is HPV driven and is simply a condyloma that is extremely large. Further research is required to determine the consequences on changing anal carcinoma incidence of HPV vaccination programmes.

The aetiology of anal adenocarcinomas is less clear; they can be HPV associated [136, 137] and HPV independent [138]. Some HPV‐associated examples are analogous to cervical stratified mucin‐producing carcinoma [136], displaying infiltrative nests of cells with varying amounts of intracytoplasmic mucin, peripheral nuclear palisading, prominent apoptosis and neutrophilic infiltrates. These express CK7 and p16 but not CK20 and p40. Other cases simulate high‐risk HPV‐related endocervical adenocarcinoma [137], showing papillary fronds of mucinous or mucin‐poor cells with plentiful apically arranged apoptotic bodies. This pattern shows expression of CK7 and p16, variable CDX2 and scant CK20 immunoreactivity. Recently characterised HPV‐independent primary perianal intestinal‐type adenocarcinoma [138, 139] and a pagetoid precursor of it are found in older adults and show an intestinal phenotype (CDX2, CK20+) and frequent *TP53* and *ERBB2* mutations with *MYC* amplification, which differ from the profile of primary extra‐mammary Paget disease (*PIK3CA* and *KMT2C* mutations), anal duct carcinoma (CK7 and MUC5 positive and CK20/CDX2 negative) [140, 141] or colorectal adenocarcinoma (*APC*, *KRAS*, *BRAF* mutations).

Adenocarcinomas are staged according to the same criteria as SCC. However, this size‐dependent stage stratification is not fully established for adenocarcinomas. Rather, recently staging based on tumour spread like rectal adenocarcinoma is reported to better discriminate patients' survival [127].

### Neuroendocrine tumours

Neuroendocrine tumours (NETs) are considered rare cancers, that can occur in almost all organs. There are longstanding aspirations to unify NET classification across the body [142] and there would be clear benefits in grading all NETs of all primary sites using the same systems, which is why all gastrointestinal NETs are described in one chapter in the new edition of the WCT of the digestive system [5]. Currently, grading is based on the mitotic count and Ki67 proliferative index [143]. Outside the gastrointestinal tract, in medullary thyroid carcinoma and pulmonary carcinoid/NET, tumour necrosis is integrated into grading [144, 145, 146]. While there is no evidence to the contrary, the evidence supporting the integration of tumour necrosis into grading schemes for gastrointestinal NETs is less strong – and mostly confined to pancreatic NETs [147]. To address this knowledge gap without overstating the current knowledge base, it is now recommended that tumour necrosis be reported alongside the grade, rather than as part of the grade; for example, grade 2 NET positive for tumour necrosis or grade 3 NET negative for tumour necrosis.

It is important to separate true tumour necrosis from infarct‐like necrosis, which is disregarded for grading purposes at other organs and therefore is currently disregarded in the gastrointestinal tract [141, 144, 145, 146]. While there is evidence that there is fair to good interobserver concordance when this distinction is made in other organs [145, 146], and some evidence from a single study that this distinction is highly reproducible in pancreatic NETs [147], there is currently insufficient evidence to conclude that this distinction can be made reproducibly across all gastrointestinal NETs.

**Figure 5:**
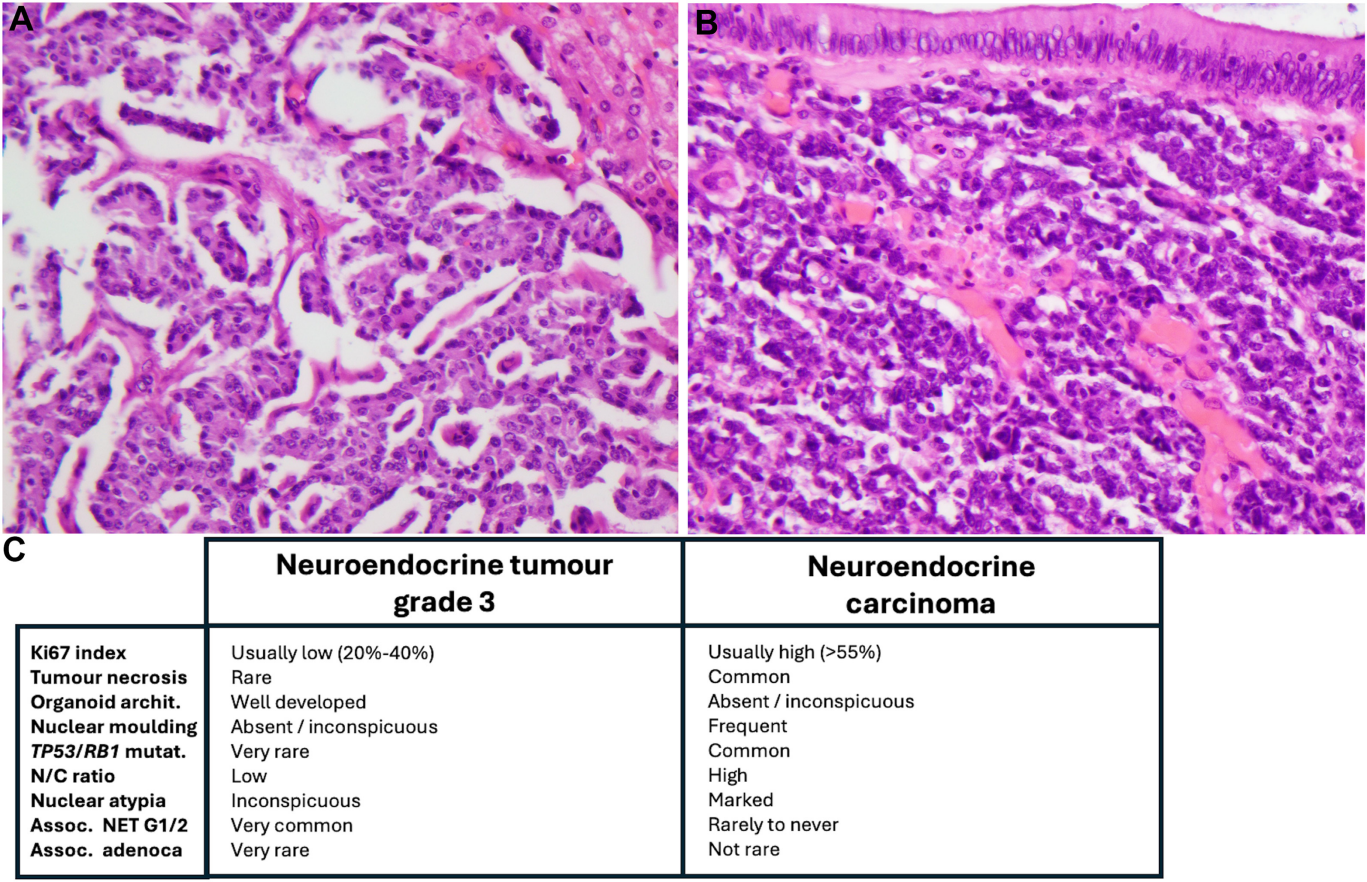
Overview of the differences between grade 3 neuroendocrine tumour (NET) and neuroendocrine carcinoma (NEC). The distinction between G3 NET and NEC may be subjective and somewhat prone to interobserver discordance. It is based not on one feature but a constellation of features. In this instance, both the tumours in panels (A) and (B) are positive for neuroendocrine markers and have a ki67 proliferative index of approximately 40%. In panel (A) (a liver metastasis from a G3 NET), the tumour maintains a trabecular and nested architecture, lacks nuclear moulding and shows less cytological atypia. In panel (B) (a NEC from the colon), the tumour grows as sheets of malignant cells, shows subtle nuclear moulding and demonstrates greater nuclear atypia. (C) Comparison of the Ki67 index, tumour necrosis, organoid architecture, nuclear moulding, *TP53/RB1* mutation status, nuclear/cytoplasmic (N/C) ratio, nuclear atypia, association with NET G1/2 and association with adenocarcinoma between G3 NET and NEC. adenoca, adenocarcinoma; archit., architecture; Assoc., associated with; mutat., mutation.

A recurrent problem in daily diagnostics is the distinction between grade 3 NETs (those low‐grade NETs with a Ki67 of greater than 20%) and neuroendocrine carcinomas (NECs) (Figure 5). The 6th edition of WCT [5] explains the criteria used to make this distinction, which are based on a range of factors including cellular atypia, architectural disarray and the presence or absence of pre‐existing low grade requirements. Despite this distinction forming a cornerstone of neuroendocrine neoplasia classification, there is regrettably little evidence that it can be made reliably and reproducibly in tumours with intermediate features between G3 NETs and NECs. Although very rare, there is increasing evidence that NETs may transform into tumours that morphologically and molecularly resemble NECs – for example, by developing increased cytological atypia, a sheet‐like architecture and accumulating events such as *RB1* and *TP53* mutations [148, 149, 150]. Although there is good evidence that these changes are associated with adverse outcomes, there is still uncertainty whether these should be considered ‘G3 NET with NEC‐like morphology’ or ‘NEC arising from NET’. In view of limited evidence to support either approach, the 6th edition of the WCT acknowledges that either term is acceptable, but more research is required.

There has been a dramatic increase in the incidence of diagnosis of gastric NETs over the last two decades [151], with a potential link to widespread use of proton pump inhibitors [152]. However, it still remains unclear whether this increased rate of diagnosis reflects a true increase in incidence or is entirely attributable to greater access to endoscopy leading to an increase in the diagnosis of asymptomatic tumours [151]. In addition, two new classes of histamine‐producing enterochromaffin‐like‐cell NETs are recognised—type 4 (associated with germline *ATP4A* mutation) and type 5 (associated with long‐term proton pump inhibitor use). While lymph node metastases have been reported in type 4 gastric NETs [153], based on limited evidence, they are thought to have a very good prognosis [154, 155]. Similarly, while there is some evidence of an excellent prognosis in type 5 gastric NETs [156], it is unclear whether this excellent prognosis applies to all gastric NETs arising in individuals with a history of more than 5 years of proton pump inhibitor use. After all, some of these tumours must represent sporadic type 3 gastric NETs coincidentally discovered in individuals taking proton pump inhibitors.

### Undifferentiated carcinomas of the gastrointestinal tract

In the 6th edition of the WCT, we have tried to clarify the topic of undifferentiated carcinomas. We now recognise tumours with deficiencies of the SWI/SNF chromatin remodelling complex attributable to somatic mutations in the *ARID1A*, *SMARCA2*, *SMARCA4* and *SMARCB1* tumour suppressor genes [157, 158, 159, 160, 161]. It was initially suspected that these high‐grade undifferentiated malignancies could represent a distinct class of neoplasia related to small cell carcinoma of the ovary of hypercalcaemic type [162], However, based initially on work in the thorax [163], which was subsequently expanded to the gastrointestinal tract [159, 164], it is now recognised that the overwhelming majority of these malignancies arise from progressive dedifferentiation of conventional carcinomas under a repeated mutagenic influence. Although poorly differentiated or rhabdoid morphology are clues to SWI/SNF deficiency, SWI/SNF complex mutations may also be found in morphologically classical carcinomas [159, 164].

Many of undifferentiated carcinomas show morphological and phenotypical mesenchymal differentiation with hot immune‐microenvironment and PD‐L1 overexpression across organs [165]. The most urgent knowledge gap is the translation of these new insights into novel immune‐oncology treatment paradigms [166].

### Knowledge gaps in rare gastrointestinal cancers – a research framework

**Figure 6:**
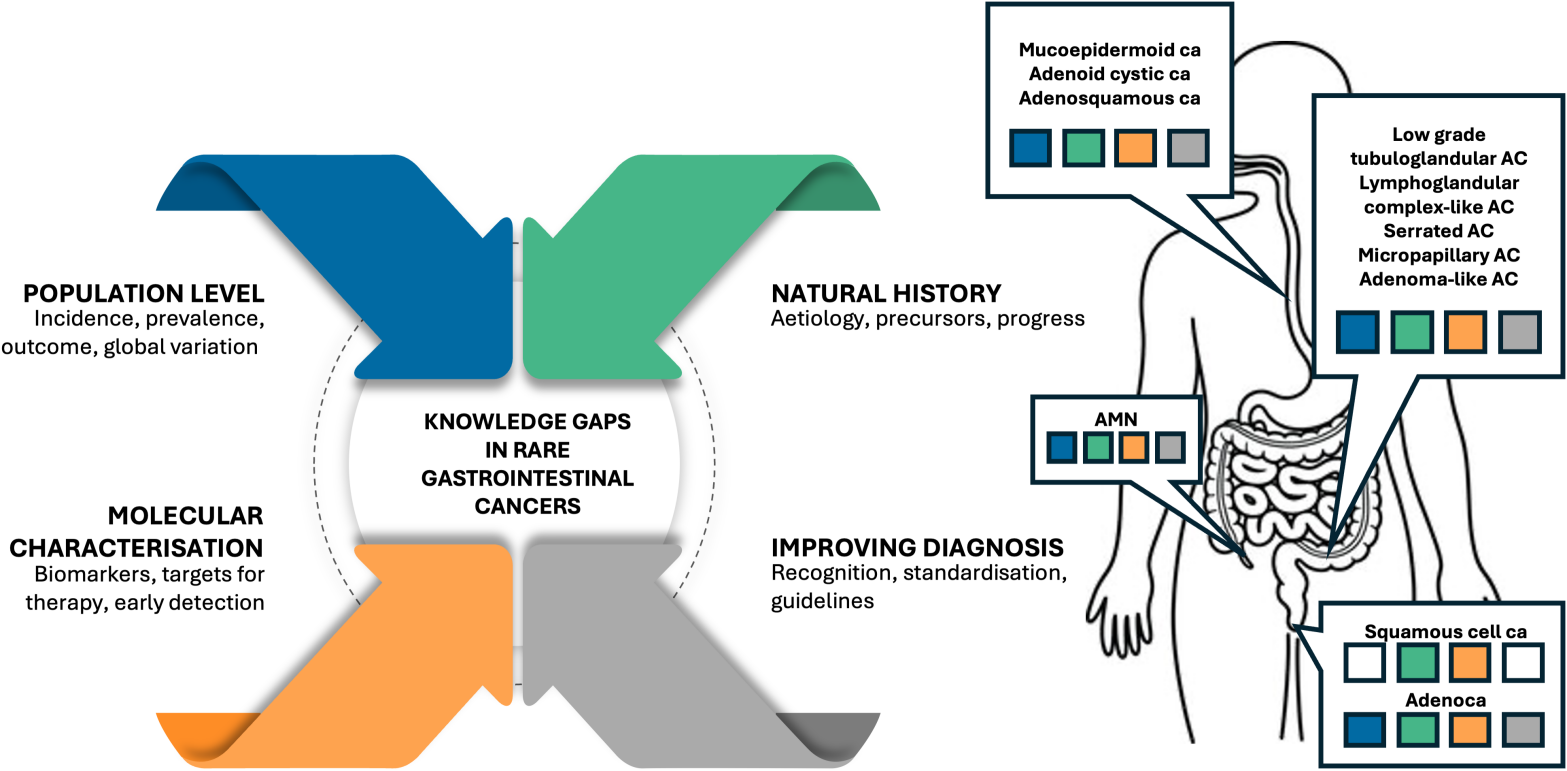
Research framework for the rare gastrointestinal cancers and knowledge gaps according to diagnosis. Knowledge gaps are indicated by the coloured boxes in the appropriate locations (white boxes represent insufficient knowledge) (diagram created with Microsoft PowerPoint). AC, adenocarcinoma; Adenoca, adenocarcinoma; AMN, appendiceal mucinous neoplasms); ca, carcinoma.

A major issue in dealing with rare cancers is making the correct diagnosis (Figure 6). For some cases, this is not difficult if they consist of common tumour types in uncommon locations (e.g. classic, intestinal type adenocarcinoma of the small bowel), and as a consequence, data at the population level are available. However, due to limited case numbers, clinical trials are almost non‐existent, and guidelines are based on other cancers: jejunoileal carcinomas are generally treated in a similar way to colorectal cancers [167] and histopathological risk factors for anal carcinomas are derived from those of colorectal cancer (ICCR in development).

However, for other rare cancer types or subtypes, robust and reproducible classification may be difficult. As a consequence, registry data are unreliable and epidemiology is less well known. From case series, relative incidences can be inferred, but these cannot be confirmed in registries since they rely on non‐expert diagnoses or registration clerk interpretation. This issue particularly hampers epidemiological studies and is likely to interfere with data obtained from clinical trials. Both treatment efficacy as well as recurrence patterns vary with tumour type and subtype [168, 169]. Correlation of histological subtypes with molecular features is well established [170, 171] but for rare cancers not yet fully explored, requiring more research. Relative treatment effects might be masked by inaccurate diagnosis or poor characterisation of rare tumour types or subtypes in reported studies or trials.

## Conclusions

Research in gastrointestinal cancers and their precursors has grown exponentially over the years, but knowledge gaps still exist and hamper efforts to decrease the burden of gastrointestinal neoplasia [171]. By defining the general questions and persistent challenges for these entities, we have designed research frameworks that focus on priority areas. While these may be conceived as practical, diagnosis‐solving issues to improve patient management, insights into the pathogenesis of precursors and rare tumours can have unexpected impact on larger groups of patients. For example, the understanding of the underlying mechanisms in Lynch syndrome has ultimately contributed to the development and implementation of immunotherapy [172].

Progress in the core research priorities for both precursor lesions and rare cancers will be reflected in future editions of the WCT. At the same time, knowledge gaps illustrated in this review emphasise the importance of global standardised classification systems, such as the current 6th edition of the WCT for the digestive system [5], since many can only be solved with collaborative registration and research efforts.

## Author contributions statement

IDN, EAM, ADP, AJG, MK and MJA all made contributions to writing the text, preparing figures and tables, and all reviewed and agreed the final manuscript.

## Data Availability

No primary data was used in the preparation of this review article.

## References

[1] Globocan, [Accessed 01 December 2025]: Available from: https://gco.iarc.who.int.

[2] Ding H , Lin J , Xu Z , *et al*. The association between organised colorectal cancer screening strategies and reduction of its related mortality: a systematic review and meta‐analysis. BMC Cancer 2024; 24 **:** 365. 38515013 10.1186/s12885-024-12054-7 PMC10958856

[3] Jodal HC , Helsingen LM , Anderson JC , *et al*. Colorectal cancer screening with faecal testing, sigmoidoscopy or colonoscopy: a systematic review and network meta‐analysis. BMJ Open 2019; 9 **:** e032773. 10.1136/bmjopen-2019-032773 PMC6797379 31578199

[4] Terasawa T , Tadano T , Abe K , *et al*. Single‐round performance of colorectal cancer screening programs: a network meta‐analysis of randomized clinical trials. BMC Med 2025; 23 **:** 110. 39985068 10.1186/s12916-025-03948-9 PMC11846209

[5] WHO Classification of Tumours Editorial Board . Digestive System Tumours (6th edn). International Agency for Research on Cancer: Lyon (France), 2025.

[6] Lam AK , Bourke MJ , Chen R , *et al*. Dataset for the reporting of carcinoma of the esophagus in resection specimens: recommendations from the international collaboration on cancer reporting. Hum Pathol 2021; 114 **:** 54–65. 33992659 10.1016/j.humpath.2021.05.003

[7] Lam AK , Nagtegaal ID , Committee for the Development of the IDfERotE , *et al*. Pathology reporting of esophagus endoscopic resections: recommendations from the international collaboration on cancer reporting. Gastroenterology 2022; 162 **:** 373–378. 34655572 10.1053/j.gastro.2021.09.069

[8] Loughrey MB , Webster F , Arends MJ , *et al*. Dataset for pathology reporting of colorectal cancer: recommendations from the international collaboration on cancer reporting (ICCR). Ann Surg 2022; 275 **:** e549–e561. 34238814 10.1097/SLA.0000000000005051 PMC8820778

[9] Rosty C , Webster F , Nagtegaal ID , *et al*. Pathology reporting of colorectal local excision specimens: recommendations from the international collaboration on cancer reporting (ICCR). Gastroenterology 2021; 161 **:** 382–387. 33961885 10.1053/j.gastro.2021.04.066

[10] Shi C , Badgwell BD , Grabsch HI , *et al*. Data set for reporting carcinoma of the stomach in gastrectomy. Arch Pathol Lab Med 2022; 146 **:** 1072–1083. 34919649 10.5858/arpa.2021-0225-OA

[11] Shi C , Webster F , Nagtegaal ID , *et al*. Pathology reporting of gastric endoscopic resections: recommendations from the international collaboration on cancer reporting. Gastroenterology 2023; 164 **:** 1039–1043. 34774830 10.1053/j.gastro.2021.11.010

[12] Marques de Sa I , Marcos P , Sharma P , *et al*. The global prevalence of Barrett's esophagus: a systematic review of the published literature. United European Gastroenterol J 2020; 8 **:** 1086–1105. 10.1177/2050640620939376 PMC7724547 32631176

[13] Eusebi LH , Cirota GG , Zagari RM , *et al*. Global prevalence of Barrett's oesophagus and oesophageal cancer in individuals with gastro‐oesophageal reflux: a systematic review and meta‐analysis. Gut 2021; 70 **:** 456–463. 32732370 10.1136/gutjnl-2020-321365

[14] Desai TK , Krishnan K , Samala N , *et al*. The incidence of oesophageal adenocarcinoma in non‐dysplastic Barrett's oesophagus: a meta‐analysis. Gut 2012; 61 **:** 970–976. 21997553 10.1136/gutjnl-2011-300730

[15] Duits LC , van der Wel MJ , Cotton CC , *et al*. Patients with Barrett's esophagus and confirmed persistent low‐grade dysplasia are at increased risk for progression to neoplasia. Gastroenterology 2017; 152 **:** 993–1001.e1. 28012849 10.1053/j.gastro.2016.12.008

[16] Skacel M , Petras RE , Gramlich TL , *et al*. The diagnosis of low‐grade dysplasia in Barrett's esophagus and its implications for disease progression. Am J Gastroenterol 2000; 95 **:** 3383–3387. 11151865 10.1111/j.1572-0241.2000.03348.x

[17] Clermont M , Falk GW . Clinical guidelines update on the diagnosis and Management of Barrett's esophagus. Dig Dis Sci 2018; 63 **:** 2122–2128. 29671159 10.1007/s10620-018-5070-z

[18] Saftoiu A , Hassan C , Areia M , *et al*. Role of gastrointestinal endoscopy in the screening of digestive tract cancers in Europe: European Society of Gastrointestinal Endoscopy (ESGE) position statement. Endoscopy 2020; 52 **:** 293–304. 32052404 10.1055/a-1104-5245

[19] Li H , Teng Y , Yan X , *et al*. Profiles and findings of population‐based esophageal cancer screening with endoscopy in China: systematic review and meta‐analysis. JMIR Public Health Surveill 2023; 9 **:** e45360. 37261899 10.2196/45360 PMC10273033

[20] Xia C , Li H , Xu Y , *et al*. Effect of an endoscopy screening on upper gastrointestinal cancer mortality: a community‐based multicenter cluster randomized clinical trial. Gastroenterology 2025; 168 **:** 725–740. 39706350 10.1053/j.gastro.2024.11.025

[21] Cottreau J , Gruchy S , Kamionek M , *et al*. Prevalence of oesophageal epidermoid metaplasia in 1048 consecutive patients and 58 patients with squamous neoplasms. Histopathology 2016; 68 **:** 988–995. 26426946 10.1111/his.12886

[22] Kamboj AK , Gibbens YY , Hagen CE , *et al*. Esophageal epidermoid metaplasia: clinical characteristics and risk of esophageal squamous neoplasia. Am J Gastroenterol 2021; 116 **:** 1533–1536. 33734117 10.14309/ajg.0000000000001225

[23] Singhi AD , Arnold CA , Crowder CD , *et al*. Esophageal leukoplakia or epidermoid metaplasia: a clinicopathological study of 18 patients. Mod Pathol 2014; 27 **:** 38–43. 23765246 10.1038/modpathol.2013.100

[24] Ezoe Y , Fujii S , Muto M , *et al*. Epidermoid metaplasia of the esophagus: endoscopic feature and differential diagnosis. Hepatogastroenterology 2011; 58 **:** 809–813. 21830395

[25] Taggart MW , Rashid A , Ross WA , *et al*. Oesophageal hyperkeratosis: clinicopathological associations. Histopathology 2013; 63 **:** 463–473. 23879628 10.1111/his.12195

[26] Singhi AD , Arnold CA , Lam‐Himlin DM , *et al*. Targeted next‐generation sequencing supports epidermoid metaplasia of the esophagus as a precursor to esophageal squamous neoplasia. Mod Pathol 2017; 30 **:** 1613–1621. 28731047 10.1038/modpathol.2017.73

[27] Pomenti SF , Flashner SP , Del Portillo A , *et al*. Clinical and biological perspectives on noncanonical esophageal squamous cell carcinoma in rare subtypes. Am J Gastroenterol 2024; 119 **:** 2376–2388. 39166765 10.14309/ajg.0000000000003041

[28] Dinis‐Ribeiro M , Libanio D , Uchima H , *et al*. Management of epithelial precancerous conditions and early neoplasia of the stomach (MAPS III): European Society of Gastrointestinal Endoscopy (ESGE), European helicobacter and microbiota study group (EHMSG) and European Society of Pathology (ESP) guideline update 2025. Endoscopy 2025; 57 **:** 504–554. 40112834 10.1055/a-2529-5025

[29] Abraham SC , Park SJ , Lee JH , *et al*. Genetic alterations in gastric adenomas of intestinal and foveolar phenotypes. Mod Pathol 2003; 16 **:** 786–795. 12920223 10.1097/01.MP.0000080349.37658.5E

[30] Kato M , Nishida T , Tsutsui S , *et al*. Endoscopic submucosal dissection as a treatment for gastric noninvasive neoplasia: a multicenter study by Osaka University ESD study group. J Gastroenterol 2011; 46 **:** 325–331. 21107615 10.1007/s00535-010-0350-1

[31] Park DY , Srivastava A , Kim GH , *et al*. Adenomatous and foveolar gastric dysplasia: distinct patterns of mucin expression and background intestinal metaplasia. Am J Surg Pathol 2008; 32 **:** 524–533. 18300795 10.1097/PAS.0b013e31815b890e

[32] Sugai T , Uesugi N , Habano W , *et al*. The clinicopathological and molecular features of sporadic gastric foveolar type neoplasia. Virchows Arch 2020; 477 **:** 835–844. 32533343 10.1007/s00428-020-02846-0 PMC7683467

[33] Naka T , Hashimoto T , Cho H , *et al*. Sporadic and familial adenomatous polyposis‐associated foveolar‐type adenoma of the stomach. Am J Surg Pathol 2023; 47 **:** 91–101. 35968980 10.1097/PAS.0000000000001949

[34] Baptista D , Fernandes M , Garrido M , *et al*. Gastric polyps in familial adenomatous polyposis Portuguese patients: the first Western cohort with Asian features. Pathobiology 2024; 91 **:** 196–204. 37852192 10.1159/000534571

[35] Vieth M , Kushima R , Borchard F , *et al*. Pyloric gland adenoma: a clinico‐pathological analysis of 90 cases. Virchows Arch 2003; 442 **:** 317–321. 12715167 10.1007/s00428-002-0750-6

[36] Chen ZM , Scudiere JR , Abraham SC , *et al*. Pyloric gland adenoma: an entity distinct from gastric foveolar type adenoma. Am J Surg Pathol 2009; 33 **:** 186–193. 18830123 10.1097/PAS.0b013e31817d7ff4

[37] Vieth M , Montgomery EA . Some observations on pyloric gland adenoma: an uncommon and long ignored entity! J Clin Pathol 2014; 67 **:** 883–890. 25092673 10.1136/jclinpath-2014-202553

[38] Matsubara A , Sekine S , Kushima R , *et al*. Frequent GNAS and KRAS mutations in pyloric gland adenoma of the stomach and duodenum. J Pathol 2013; 229 **:** 579–587. 23208952 10.1002/path.4153

[39] Setia N , Wanjari P , Yassan L , *et al*. Next‐generation sequencing identifies 2 genomically distinct groups among pyloric gland adenomas. Hum Pathol 2020; 97 **:** 103–111. 31783043 10.1016/j.humpath.2019.11.004

[40] Hackeng WM , Montgomery EA , Giardiello FM , *et al*. Morphology and genetics of pyloric gland adenomas in familial adenomatous polyposis. Histopathology 2017; 70 **:** 549–557. 27767239 10.1111/his.13105 PMC5300963

[41] Hashimoto T , Ogawa R , Matsubara A , *et al*. Familial adenomatous polyposis‐associated and sporadic pyloric gland adenomas of the upper gastrointestinal tract share common genetic features. Histopathology 2015; 67 **:** 689–698. 25832318 10.1111/his.12705

[42] Choi WT , Brown I , Ushiku T , *et al*. Gastric pyloric gland adenoma: a multicentre clinicopathological study of 67 cases. Histopathology 2018; 72 **:** 1007–1014. 29278427 10.1111/his.13460

[43] Ushiku T , Kunita A , Kuroda R , *et al*. Oxyntic gland neoplasm of the stomach: expanding the spectrum and proposal of terminology. Mod Pathol 2020; 33 **:** 206–216. 31375767 10.1038/s41379-019-0338-1

[44] Shinozaki‐Ushiku A , Koinuma D , Nakayama A , *et al*. Diffuse cyclin D1 and SPINK1 expression in gastric oxyntic gland neoplasms: promising diagnostic markers identified using spatial transcriptome analysis. Mod Pathol 2025; 38 **:** 100719. 39863113 10.1016/j.modpat.2025.100719

[45] Zhai Z , Hu W , Huang Z , *et al*. Gastric adenocarcinoma of the fundic gland type: a review of the literature. JGH Open 2023; 7 **:** 812–825. 38162862 10.1002/jgh3.13014 PMC10757499

[46] Mohammed A , Trujillo S , Ghoneim S , *et al*. Small bowel adenocarcinoma: a nationwide population‐based study. J Gastrointest Cancer 2023; 54 **:** 67–72. 35001295 10.1007/s12029-021-00653-7

[47] Pedersen KS , Raghav K , Overman MJ . Small bowel adenocarcinoma: etiology, presentation, and molecular alterations. J Natl Compr Canc Netw 2019; 17 **:** 1135–1141. 31487680 10.6004/jnccn.2019.7344

[48] Siegel RL , Giaquinto AN , Jemal A . Cancer statistics, 2024. CA Cancer J Clin 2024; 74 **:** 12–49. 38230766 10.3322/caac.21820

[49] Ushiku T , Arnason T , Fukayama M , *et al*. Extra‐ampullary duodenal adenocarcinoma. Am J Surg Pathol 2014; 38 **:** 1484–1493. 25310836 10.1097/PAS.0000000000000278

[50] Adsay V , Ohike N , Tajiri T , *et al*. Ampullary region carcinomas: definition and site specific classification with delineation of four clinicopathologically and prognostically distinct subsets in an analysis of 249 cases. Am J Surg Pathol 2012; 36 **:** 1592–1608. 23026934 10.1097/PAS.0b013e31826399d8

[51] Onkendi EO , Boostrom SY , Sarr MG , *et al*. 15‐year experience with surgical treatment of duodenal carcinoma: a comparison of periampullary and extra‐ampullary duodenal carcinomas. J Gastrointest Surg 2012; 16 **:** 682–691. 22350721 10.1007/s11605-011-1808-z

[52] Uijterwijk BA , Lemmers DH , Ghidini M , *et al*. The five periampullary cancers, not just different siblings but different families: an international multicenter cohort study. Ann Surg Oncol 2024; 31 **:** 6157–6169. 38888860 10.1245/s10434-024-15555-8

[53] Tarcan ZC , Esmer R , Akar KE , *et al*. Intra‐ampullary papillary tubular neoplasm (IAPN): clinicopathologic analysis of 72 cases highlights the distinctive characteristics of a poorly recognized entity. Am J Surg Pathol 2024; 48 **:** 1093–1107. 38938087 10.1097/PAS.0000000000002275

[54] Russell TB , Labib PL , Denson J , *et al*. Predictors of actual five‐year survival and recurrence after pancreatoduodenectomy for ampullary adenocarcinoma: results from an international multicentre retrospective cohort study. HPB (Oxford) 2023; 25 **:** 788–797. 37149485 10.1016/j.hpb.2023.03.010

[55] Xue Y , Vanoli A , Balci S , *et al*. Non‐ampullary‐duodenal carcinomas: clinicopathologic analysis of 47 cases and comparison with ampullary and pancreatic adenocarcinomas. Mod Pathol 2017; 30 **:** 255–266. 27739441 10.1038/modpathol.2016.174

[56] Buchanan DD , Clendenning M , Zhuoer L , *et al*. Lack of evidence for germline RNF43 mutations in patients with serrated polyposis syndrome from a large multinational study. Gut 2017; 66 **:** 1170–1172. 27582512 10.1136/gutjnl-2016-312773

[57] Quintana I , Mejias‐Luque R , Terradas M , *et al*. Evidence suggests that germline RNF43 mutations are a rare cause of serrated polyposis. Gut 2018; 67 **:** 2230–2232. 29330307 10.1136/gutjnl-2017-315733

[58] Yan HHN , Lai JCW , Ho SL , *et al*. RNF43 germline and somatic mutation in serrated neoplasia pathway and its association with BRAF mutation. Gut 2017; 66 **:** 1645–1656. 27329244 10.1136/gutjnl-2016-311849

[59] Clendenning M , Young JP , Walsh MD , *et al*. Germline mutations in the polyposis‐associated genes BMPR1A, SMAD4, PTEN, MUTYH and GREM1 are not common in individuals with serrated polyposis syndrome. PLoS One 2013; 8 **:** e66705. 23805267 10.1371/journal.pone.0066705 PMC3689730

[60] Soares de Lima Y , Arnau‐Collell C , Munoz J , *et al*. Germline mutations in WNK2 could be associated with serrated polyposis syndrome. J Med Genet 2023; 60 **:** 557–567. 36270769 10.1136/jmg-2022-108684 PMC10313964

[61] Toma C , Diaz‐Gay M , Soares de Lima Y , *et al*. Identification of a novel candidate gene for serrated polyposis syndrome germline predisposition by performing linkage analysis combined with whole‐exome sequencing. Clin Transl Gastroenterol 2019; 10 **:** e00100. 31663907 10.14309/ctg.0000000000000100 PMC6919450

[62] Boparai KS , Dekker E , Van Eeden S , *et al*. Hyperplastic polyps and sessile serrated adenomas as a phenotypic expression of MYH‐associated polyposis. Gastroenterology 2008; 135 **:** 2014–2018. 19013464 10.1053/j.gastro.2008.09.020

[63] Borowsky J , Setia N , Rosty C , *et al*. Spectrum of gastrointestinal tract pathology in a multicenter cohort of 43 Cowden syndrome patients. Mod Pathol 2019; 32 **:** 1814–1822. 31273317 10.1038/s41379-019-0316-7

[64] Jaeger E , Leedham S , Lewis A , *et al*. Hereditary mixed polyposis syndrome is caused by a 40‐kb upstream duplication that leads to increased and ectopic expression of the BMP antagonist GREM1. Nat Genet 2012; 44 **:** 699–703. 22561515 10.1038/ng.2263 PMC4594751

[65] Anthony E , Reece JC , Milanzi E , *et al*. Body mass index, sex, non‐steroidal anti‐inflammatory drug medications, smoking and alcohol are differentially associated with World Health Organization criteria and colorectal cancer risk in people with serrated polyposis syndrome: an Australian case‐control study. BMC Gastroenterol 2022; 22 **:** 489. 36435745 10.1186/s12876-022-02557-7 PMC9701413

[66] Boparai KS , Dekker E , Polak MM , *et al*. A serrated colorectal cancer pathway predominates over the classic WNT pathway in patients with hyperplastic polyposis syndrome. Am J Pathol 2011; 178 **:** 2700–2707. 21641392 10.1016/j.ajpath.2011.02.023 PMC3124356

[67] Rosty C , Walsh MD , Walters RJ , *et al*. Multiplicity and molecular heterogeneity of colorectal carcinomas in individuals with serrated polyposis. Am J Surg Pathol 2013; 37 **:** 434–442. 23211288 10.1097/PAS.0b013e318270f748 PMC3567207

[68] Wijnands AM , de Jong ME , Lutgens M , *et al*. Prognostic factors for advanced colorectal neoplasia in inflammatory bowel disease: systematic review and meta‐analysis. Gastroenterology 2021; 160 **:** 1584–1598. 33385426 10.1053/j.gastro.2020.12.036

[69] Murthy SK , Feuerstein JD , Nguyen GC , *et al*. AGA clinical practice update on endoscopic surveillance and Management of Colorectal Dysplasia in inflammatory bowel diseases: expert review. Gastroenterology 2021; 161 **:** 1043–1051.e4. 34416977 10.1053/j.gastro.2021.05.063

[70] Alpert L , Setia N , Ko HM , *et al*. Interobserver agreement and the impact of mentorship on the diagnosis of inflammatory bowel disease‐associated dysplasia among subspecialist gastrointestinal pathologists. Virchows Arch 2021; 478 **:** 1061–1069. 33392796 10.1007/s00428-020-02998-z

[71] Jimeno M , Domingo A , Salas I , *et al*. Pathologist experience and concordance in the diagnosis of dysplasia in long‐standing inflammatory bowel disease. Am J Surg Pathol 2020; 44 **:** 955–961. 32235151 10.1097/PAS.0000000000001475

[72] Schlemper RJ , Riddell RH , Kato Y , *et al*. The Vienna classification of gastrointestinal epithelial neoplasia. Gut 2000; 47 **:** 251–255. 10896917 10.1136/gut.47.2.251 PMC1728018

[73] Riddell RH , Goldman H , Ransohoff DF , *et al*. Dysplasia in inflammatory bowel disease: standardized classification with provisional clinical applications. Hum Pathol 1983; 14 **:** 931–968. 6629368 10.1016/s0046-8177(83)80175-0

[74] Nasreddin N , Jansen M , Loughrey MB , *et al*. Poor diagnostic reproducibility in the identification of nonconventional dysplasia in colitis impacts the application of histologic stratification tools. Mod Pathol 2024; 37 **:** 100419. 38158125 10.1016/j.modpat.2023.100419

[75] Choi WT , Yozu M , Miller GC , *et al*. Nonconventional dysplasia in patients with inflammatory bowel disease and colorectal carcinoma: a multicenter clinicopathologic study. Mod Pathol 2020; 33 **:** 933–943. 31822800 10.1038/s41379-019-0419-1

[76] Harpaz N , Goldblum JR , Shepherd NA , *et al*. Colorectal dysplasia in chronic inflammatory bowel disease: a contemporary consensus classification and interobserver study. Hum Pathol 2023; 138 **:** 49–61. 37247824 10.1016/j.humpath.2023.05.008

[77] Zhou RW , Harpaz N , Itzkowitz SH , *et al*. Molecular mechanisms in colitis‐associated colorectal cancer. Oncogene 2023; 12 **:** 48. 10.1038/s41389-023-00492-0 PMC10603140 37884500

[78] Din S , Wong K , Mueller MF , *et al*. Mutational analysis identifies therapeutic biomarkers in inflammatory bowel disease‐associated colorectal cancers. Clin Cancer Res 2018; 24 **:** 5133–5142. 29950348 10.1158/1078-0432.CCR-17-3713 PMC6193541

[79] Leedham SJ , Graham TA , Oukrif D , *et al*. Clonality, founder mutations, and field cancerization in human ulcerative colitis‐associated neoplasia. Gastroenterology 2009; 136 **:** 542–550.e6. 19103203 10.1053/j.gastro.2008.10.086

[80] Choi WT , Salomao M , Zhao L , *et al*. Hypermucinous, goblet cell‐deficient and crypt cell Dysplasias in inflammatory bowel disease are often associated with flat/invisible endoscopic appearance and advanced neoplasia on follow‐up. J Crohns Colitis 2022; 16 **:** 98–108. 34232295 10.1093/ecco-jcc/jjab120

[81] Lee H , Rabinovitch PS , Mattis AN , *et al*. Non‐conventional dysplasia in inflammatory bowel disease is more frequently associated with advanced neoplasia and aneuploidy than conventional dysplasia. Histopathology 2021; 78 **:** 814–830. 33155325 10.1111/his.14298

[82] Al Bakir I , Curius K , Cressell GD , *et al*. Low‐coverage whole genome sequencing of low‐grade dysplasia strongly predicts advanced neoplasia risk in ulcerative colitis. Gut 2025; 74 **:** 740–751. 39880602 10.1136/gutjnl-2024-333353 PMC12013573

[83] Darragh TM , Colgan TJ , Cox JT , *et al*. The lower anogenital squamous terminology standardization project for HPV‐associated lesions: background and consensus recommendations from the College of American Pathologists and the American Society for Colposcopy and Cervical Pathology. Arch Pathol Lab Med 2012; 136 **:** 1266–1297. 22742517 10.5858/arpa.LGT200570

[84] Mendez‐Martinez R , Rivera‐Martinez NE , Crabtree‐Ramirez B , *et al*. Multiple human papillomavirus infections are highly prevalent in the anal canal of human immunodeficiency virus‐positive men who have sex with men. BMC Infect Dis 2014; 14 **:** 671. 25510243 10.1186/s12879-014-0671-4 PMC4272559

[85] Cameron RL , Cuschieri K , Pollock KGJ . Baseline HPV prevalence in rectal swabs from men attending a sexual health clinic in Scotland: assessing the potential impact of a selective HPV vaccination programme for men who have sex with men. Sex Transm Infect 2020; 96 **:** 55–57. 30636708 10.1136/sextrans-2018-053668

[86] Rantshabeng PS , Moyo S , Moraka NO , *et al*. Prevalence of oncogenic human papillomavirus genotypes in patients diagnosed with anogenital malignancies in Botswana. BMC Infect Dis 2017; 17 **:** 731. 29178840 10.1186/s12879-017-2832-8 PMC5702116

[87] Pinto A , Singhi AD , Voltaggio L , *et al*. TP53 wild‐type, human papillomavirus‐independent anal growth/(intra)epithelial lesion (ANGEL): a potential precursor of anal squamous cell carcinoma. Mod Pathol 2025; 38 **:** 100721. 39863109 10.1016/j.modpat.2025.100721

[88] Wei F , Gaisa MM , D'Souza G , *et al*. Epidemiology of anal human papillomavirus infection and high‐grade squamous intraepithelial lesions in 29 900 men according to HIV status, sexuality, and age: a collaborative pooled analysis of 64 studies. Lancet HIV 2021; 8 **:** e531–e543. 34339628 10.1016/S2352-3018(21)00108-9 PMC8408042

[89] Nguyen ED , Wang D , Lauwers GY , *et al*. Increased histologic inflammation is an independent risk factor for nonconventional dysplasia in ulcerative colitis. Histopathology 2022; 81 **:** 644–652. 35942654 10.1111/his.14765

[90] Bahceci D , Choi WT . Recent updates and debates on basal crypt dysplasia, serrated epithelial change, and p53 immunostaining in inflammatory bowel disease. Hum Pathol 2025; 169 **:** 105959. 41159918 10.1016/j.humpath.2025.105959

[91] Wani S , Zhou MJ , Sawas T , *et al*. AGA clinical practice guideline on surveillance of Barrett's esophagus. Gastroenterology 2025; 169 **:** 1184–1231. 41125322 10.1053/j.gastro.2025.09.012

[92] Chornenkyy Y , Vyas M , Deshpande V . The future is now: advancing p53 immunohistochemistry in Barrett's oesophagus and its implication for the everyday pathologist. Histopathology 2026; 88 **:** 380–401. 40223170 10.1111/his.15442

[93] Chromy D , Aigner F , Becker JC , *et al*. German‐Austrian guideline on screening for anal dysplasia and anal carcinoma in people living with HIV. J Dtsch Dermatol Ges 2025; 23 **:** 1025–1040. 40320909 10.1111/ddg.15719 PMC12338428

[94] Loughrey MB , Shepherd NA . Anal and perianal preneoplastic lesions. Gastroenterol Clin North Am 2024; 53 **:** 201–220. 38280748 10.1016/j.gtc.2023.09.007

[95] US NIH National Cancer Institute . Dictionary of Cancer Terms, Available from: https://www.cancer.gov/publications/dictionaries/cancer-terms/def/rare-cancer.

[96] Rarecarenet: advancing care for rare cancers across Europe, Available from: rarecarenet.eu.

[97] Chen S , Chen Y , Yang J , *et al*. Primary mucoepidermoid carcinoma of the esophagus. J Thorac Oncol 2011; 6 **:** 1426–1431. 21587086 10.1097/JTO.0b013e31821cfb96

[98] Chen SB , Weng HR , Wang G , *et al*. Primary adenosquamous carcinoma of the esophagus. World J Gastroenterol 2013; 19 **:** 8382–8390. 24363531 10.3748/wjg.v19.i45.8382 PMC3857463

[99] Karaoglanoglu N , Eroglu A , Turkyilmaz A , *et al*. Oesophageal adenoid cystic carcinoma and its management options. Int J Clin Pract 2005; 59 **:** 1101–1103. 16115189 10.1111/j.1742-1241.2005.00556.x

[100] Ni PZ , Yang YS , Hu WP , *et al*. Primary adenosquamous carcinoma of the esophagus: an analysis of 39 cases. J Thorac Dis 2016; 8 **:** 2689–2696. 27867543 10.21037/jtd.2016.09.59 PMC5107504

[101] Petursson SR . Adenoid cystic carcinoma of the esophagus. Complete response to combination chemotherapy. Cancer 1986; 57 **:** 1464–1467. 3004692 10.1002/1097-0142(19860415)57:8<1464::aid-cncr2820570805>3.0.co;2-f

[102] Sawada G , Moon J , Saito A , *et al*. A case of adenoid cystic carcinoma of the esophagus. Surg Case Rep 2015; 1 **:** 119. 26943443 10.1186/s40792-015-0122-5 PMC4662665

[103] Zhou Y , Zang Y , Xiang J , *et al*. Adenoid cystic carcinoma of the cardia: report of a rare case and review of the Chinese literature. Oncol Lett 2014; 8 **:** 726–730. 25013491 10.3892/ol.2014.2153 PMC4081280

[104] Lam KY , Loke SL , Ma LT . Histochemistry of mucin secreting components in mucoepidermoid and adenosquamous carcinoma of the oesophagus. J Clin Pathol 1993; 46 **:** 1011–1015. 7504701 10.1136/jcp.46.11.1011 PMC501684

[105] van Rees BP , Rouse RW , de Wit MJ , *et al*. Molecular evidence for the same clonal origin of both components of an adenosquamous Barrett carcinoma. Gastroenterology 2002; 122 **:** 784–788. 11875011 10.1053/gast.2002.31903

[106] Milne AN , Carvalho R , van Rees BP , *et al*. Do collision tumors of the gastroesophageal junction exist? A molecular analysis. Am J Surg Pathol 2004; 28 **:** 1492–1498. 15489653 10.1097/01.pas.0000138184.74496.4d

[107] North JP , McCalmont TH , Fehr A , *et al*. Detection of MYB alterations and other immunohistochemical markers in primary cutaneous adenoid cystic carcinoma. Am J Surg Pathol 2015; 39 **:** 1347–1356. 26076064 10.1097/PAS.0000000000000463

[108] Batra H , Bose PSC , Ding Y , *et al*. MYB expression by immunohistochemistry is highly specific and sensitive for detection of solid variant of adenoid cystic carcinoma of the breast among all triple‐negative breast cancers. Histopathology 2024; 85 **:** 503–509. 38973399 10.1111/his.15276

[109] Japan Esophageal Society . Japanese classification of esophageal cancer, 11th edition: part II and III. Esophagus 2017; 14 **:** 37–65. 28111536 10.1007/s10388-016-0556-2 PMC5222925

[110] Holowatyj AN , Overman MJ , Votanopoulos KI , *et al*. Defining a ‘cells to society’ research framework for appendiceal tumours. Nat Rev Cancer 2025; 25 **:** 293–315. 39979656 10.1038/s41568-024-00788-2 PMC12427072

[111] Carr NJ , McCarthy WF , Sobin LH . Epithelial noncarcinoid tumors and tumor‐like lesions of the appendix. A clinicopathologic study of 184 patients with a multivariate analysis of prognostic factors. Cancer 1995; 75 **:** 757–768. 7828125 10.1002/1097-0142(19950201)75:3<757::aid-cncr2820750303>3.0.co;2-f

[112] Yantiss RK , Shia J , Klimstra DS , *et al*. Prognostic significance of localized extra‐appendiceal mucin deposition in appendiceal mucinous neoplasms. Am J Surg Pathol 2009; 33 **:** 248–255. 18852679 10.1097/PAS.0b013e31817ec31e

[113] Pai RK , Beck AH , Norton JA , *et al*. Appendiceal mucinous neoplasms: clinicopathologic study of 116 cases with analysis of factors predicting recurrence. Am J Surg Pathol 2009; 33 **:** 1425–1439. 19641451 10.1097/PAS.0b013e3181af6067

[114] Polydorides AD , Wen X . Clinicopathologic parameters and outcomes of mucinous neoplasms confined to the appendix: a benign entity with excellent prognosis. Mod Pathol 2022; 35 **:** 1732–1739. 35676331 10.1038/s41379-022-01114-7

[115] Gonzalez RS , Carr NJ , Liao H , *et al*. High‐grade appendiceal mucinous neoplasm: clinicopathologic findings in 35 cases. Arch Pathol Lab Med 2022; 146 **:** 1471–1478. 35472721 10.5858/arpa.2021-0430-OA

[116] Dartigues P , Kepenekian V , Illac‐Vauquelin C , *et al*. Insights into the clinical prognosis of high‐grade appendiceal mucinous neoplasms. Am J Surg Pathol 2025; 49 **:** 499–507. 39975985 10.1097/PAS.0000000000002373 PMC11984543

[117] Zhu X , Salhab M , Tomaszewicz K , *et al*. Heterogeneous mutational profile and prognosis conferred by TP53 mutations in appendiceal mucinous neoplasms. Hum Pathol 2019; 85 **:** 260–269. 30458197 10.1016/j.humpath.2018.11.011

[118] Foote MB , Walch H , Chatila W , *et al*. Molecular classification of appendiceal adenocarcinoma. J Clin Oncol 2023; 41 **:** 1553–1564. 36493333 10.1200/JCO.22.01392 PMC10043565

[119] Wald AI , Pingpank JF , Ongchin M , *et al*. Targeted next‐generation sequencing improves the prognostication of patients with disseminated appendiceal mucinous neoplasms (pseudomyxoma peritonei). Ann Surg Oncol 2023; 30 **:** 7517–7526. 37314541 10.1245/s10434-023-13721-y

[120] Gibson J , Pengelly RJ , Mirandari A , *et al*. Targeted genetic sequencing analysis of 223 cases of pseudomyxoma peritonei treated by cytoreductive surgery and hyperthermic intraperitoneal chemotherapy shows survival related to GNAS and KRAS status. Cancer Med 2024; 13 **:** e70340. 39435876 10.1002/cam4.70340 PMC11494485

[121] Vink‐Borger E , Knijn N , de Bruine A , *et al*. Is it time to acknowledge intramucosal colorectal carcinoma? Histopathology 2025; 86 **:** 805–812. 39648952 10.1111/his.15389 PMC11903115

[122] Kojima M , Shimazaki H , Iwaya K , *et al*. Intramucosal colorectal carcinoma with invasion of the lamina propria: a study by the Japanese Society for Cancer of the Colon and Rectum. Hum Pathol 2017; 66 **:** 230–237. 28711649 10.1016/j.humpath.2017.04.031

[123] Akarca FG , Yozu M , Alpert L , *et al*. Non‐conventional dysplasia is frequently associated with low‐grade tubuloglandular and mucinous adenocarcinomas in inflammatory bowel disease. Histopathology 2023; 83 **:** 276–285. 37055929 10.1111/his.14922

[124] Hartman DJ , Binion D , Regueiro M , *et al*. Isocitrate dehydrogenase‐1 is mutated in inflammatory bowel disease‐associated intestinal adenocarcinoma with low‐grade tubuloglandular histology but not in sporadic intestinal adenocarcinoma. Am J Surg Pathol 2014; 38 **:** 1147–1156. 25029120 10.1097/PAS.0000000000000239

[125] Yilmaz O , Westerhoff M , Panarelli N , *et al*. Lymphoglandular complex‐like colorectal carcinoma‐a series of 20 colorectal cases, including newly reported features of malignant behavior. Am J Surg Pathol 2024; 48 **:** 70–79. 38054635 10.1097/PAS.0000000000002141

[126] Lee HE , Wu TT , Chandan VS , *et al*. Colonic adenomatous polyps involving submucosal lymphoglandular complexes: a diagnostic pitfall. Am J Surg Pathol 2018; 42 **:** 1083–1089. 29738362 10.1097/PAS.0000000000001081

[127] Troester A , Kohn J , Wang Q , *et al*. Management and staging of anal adenocarcinoma in the United States: a population‐based analysis. J Gastrointest Surg 2024; 28 **:** 519–527. 38583905 10.1016/j.gassur.2024.01.013

[128] Franklin RA , Giri S , Valasareddy P , *et al*. Comparative survival of patients with anal adenocarcinoma, squamous cell carcinoma of the anus, and rectal adenocarcinoma. Clin Colorectal Cancer 2016; 15 **:** 47–53. 26362848 10.1016/j.clcc.2015.07.007

[129] Shiels MS , Pfeiffer RM , Chaturvedi AK , *et al*. Impact of the HIV epidemic on the incidence rates of anal cancer in the United States. J Natl Cancer Inst 2012; 104 **:** 1591–1598. 23042932 10.1093/jnci/djs371 PMC3611819

[130] Lokko C , Turner J , Yoo W , *et al*. Anal squamous cell carcinoma in African Americans with and without HIV: a comparative study. J Cancer Epidemiol Treat 2015; 1 **:** 6–10. 27774311 10.24218/jcet.2015.04 PMC5074337

[131] Oehler‐Janne C , Huguet F , Provencher S , *et al*. HIV‐specific differences in outcome of squamous cell carcinoma of the anal canal: a multicentric cohort study of HIV‐positive patients receiving highly active antiretroviral therapy. J Clin Oncol 2008; 26 **:** 2550–2557. 18427149 10.1200/JCO.2007.15.2348

[132] Shiels MS , Kreimer AR , Coghill AE , *et al*. Anal cancer incidence in the United States, 1977‐2011: distinct patterns by histology and behavior. Cancer Epidemiol Biomarkers Prev 2015; 24 **:** 1548–1556. 26224796 10.1158/1055-9965.EPI-15-0044 PMC4592448

[133] van der Zee RP , Richel O , de Vries HJ , *et al*. The increasing incidence of anal cancer: can it be explained by trends in risk groups? Neth J Med 2013; 71 **:** 401–411. 24127500

[134] Soeberg MJ , Rogers K , Currow DC , *et al*. Trends in incidence and survival for anal cancer in New South Wales, Australia, 1972‐2009. Cancer Epidemiol 2015; 39 **:** 842–847. 26651444 10.1016/j.canep.2015.10.008

[135] Bouvier AM , Belot A , Manfredi S , *et al*. Trends of incidence and survival in squamous‐cell carcinoma of the anal canal in France: a population‐based study. Eur J Cancer Prev 2016; 25 **:** 182–187. 25973771 10.1097/CEJ.0000000000000163

[136] Sappenfield R , Camacho‐Cordovez F , Larman T , *et al*. Stratified mucin‐producing lesions of the anus: insights into an emerging histologic type of HPV‐driven anal neoplasia. Am J Surg Pathol 2025; 49 **:** 121–129. 39308041 10.1097/PAS.0000000000002312

[137] Voltaggio L , McCluggage WG , Iding JS , *et al*. A novel group of HPV‐related adenocarcinomas of the lower anogenital tract (vagina, vulva, and anorectum) in women and men resembling HPV‐related endocervical adenocarcinomas. Mod Pathol 2020; 33 **:** 944–952. 31857682 10.1038/s41379-019-0437-z

[138] Bahceci D , Saoud C , Isidro RA , *et al*. Perianal intestinal‐type Paget disease with and without invasion, unassociated with internal malignancy: a distinct form of primary perianal adenocarcinoma. Mod Pathol 2025; 39 **:** 100917. 41106477 10.1016/j.modpat.2025.100917 PMC13159077

[139] Gill PS , Wong N . Primary peri‐anal adenocarcinoma of intestinal type ‐ a new proposed entity. Histopathology 2018; 73 **:** 157–161. 29464744 10.1111/his.13495

[140] Hobbs CM , Lowry MA , Owen D , *et al*. Anal gland carcinoma. Cancer 2001; 92 **:** 2045–2049. 11596018 10.1002/1097-0142(20011015)92:8<2045::aid-cncr1543>3.0.co;2-v

[141] Meriden Z , Montgomery EA . Anal duct carcinoma: a report of 5 cases. Hum Pathol 2012; 43 **:** 216–220. 21820151 10.1016/j.humpath.2011.04.019

[142] Rindi G , Klimstra DS , Abedi‐Ardekani B , *et al*. A common classification framework for neuroendocrine neoplasms: an International Agency for Research on Cancer (IARC) and World Health Organization (WHO) expert consensus proposal. Mod Pathol 2018; 31 **:** 1770–1786. 30140036 10.1038/s41379-018-0110-y PMC6265262

[143] Rindi G , Mete O , Uccella S , *et al*. Overview of the 2022 WHO classification of neuroendocrine neoplasms. Endocr Pathol 2022; 33 **:** 115–154. 35294740 10.1007/s12022-022-09708-2

[144] Travis WD . Pathology and diagnosis of neuroendocrine tumors: lung neuroendocrine. Thorac Surg Clin 2014; 24 **:** 257–266. 25065926 10.1016/j.thorsurg.2014.04.001

[145] Fuchs TL , Nassour AJ , Glover A , *et al*. A proposed grading scheme for medullary thyroid carcinoma based on proliferative activity (ki‐67 and mitotic count) and coagulative necrosis. Am J Surg Pathol 2020; 44 **:** 1419–1428. 32452872 10.1097/PAS.0000000000001505 PMC7641183

[146] Xu B , Fuchs TL , Ahmadi S , *et al*. International medullary thyroid carcinoma grading system: a validated grading system for medullary thyroid carcinoma. J Clin Oncol 2022; 40 **:** 96–104. 34731032 10.1200/JCO.21.01329 PMC8683221

[147] Fuchs TL , Chou A , Ahadi M , *et al*. Necrosis is an independent predictor of disease‐free and overall survival in pancreatic well‐differentiated neuroendocrine tumours (NETs): a proposal to include it in grading systems. Pathology 2022; 54 **:** 855–862. 35934531 10.1016/j.pathol.2022.05.008

[148] Kasajima A , Pfarr N , Mayr EM , *et al*. Rapid evolution of metastases in patients with treated G3 neuroendocrine tumors associated with NEC‐like transformation and TP53 mutation. Endocr Pathol 2024; 35 **:** 313–324. 39382626 10.1007/s12022-024-09827-y PMC11659366

[149] Joseph NM , Umetsu SE , Kim GE , *et al*. Progression of low‐grade neuroendocrine tumors (NET) to high‐grade neoplasms harboring the NEC‐like Co‐alteration of RB1 and TP53. Endocr Pathol 2024; 35 **:** 325–337. 39556303 10.1007/s12022-024-09835-y PMC11659342

[150] Kanaan C , Bani MA , Ducreux M , *et al*. Diagnostic relevance of p53 and Rb status in neuroendocrine tumors G3 from different organs: an immunohistochemical study of 465 high‐grade neuroendocrine neoplasms. Virchows Arch 2025; 486 **:** 941–950. 39671088 10.1007/s00428-024-04006-0

[151] Dasari A , Shen C , Halperin D , *et al*. Trends in the incidence, prevalence, and survival outcomes in patients with neuroendocrine tumors in the United States. JAMA Oncol 2017; 3 **:** 1335–1342. 28448665 10.1001/jamaoncol.2017.0589 PMC5824320

[152] McCarthy DM . Proton pump inhibitor use, hypergastrinemia, and gastric carcinoids‐what is the relationship? Int J Mol Sci 2020; 21 **:** 662. 31963924 10.3390/ijms21020662 PMC7014182

[153] Abraham SC , Carney JA , Ooi A , *et al*. Achlorhydria, parietal cell hyperplasia, and multiple gastric carcinoids: a new disorder. Am J Surg Pathol 2005; 29 **:** 969–975. 15958864 10.1097/01.pas.0000163363.86099.9f

[154] Calvete O , Reyes J , Valdes‐Socin H , *et al*. Alterations in SLC4A2, SLC26A7 and SLC26A9 drive Acid‐Base imbalance in gastric neuroendocrine tumors and uncover a novel mechanism for a Co‐occurring Polyautoimmune scenario. Cells 2021; 10 **:** 3500. 34944008 10.3390/cells10123500 PMC8700745

[155] Sasaki Y , Abe Y , Haruma K , *et al*. Multiple gastric neuroendocrine tumors in a patient with parietal cell dysfunction and adenosine triphosphatase H^+^/K^+^ transporting subunit alpha gene variant. Clin J Gastroenterol 2024; 17 **:** 607–616. 38635098 10.1007/s12328-024-01969-0

[156] Al‐Toubah T , Pelle E , Haider M , *et al*. Association between long‐term proton pump inhibitor use and low‐risk gastric neuroendocrine tumors. Ann Surg Oncol 2026; 33 **:** 594–598. 41028638 10.1245/s10434-025-18430-2

[157] Singhi AD , Seethala RR , Nason K , *et al*. Undifferentiated carcinoma of the esophagus: a clinicopathological study of 16 cases. Hum Pathol 2015; 46 **:** 366–375. 25582499 10.1016/j.humpath.2014.11.021 PMC4384179

[158] Chang B , Sheng W , Wang L , *et al*. SWI/SNF complex‐deficient undifferentiated carcinoma of the gastrointestinal tract: clinicopathologic study of 30 cases with an emphasis on variable morphology, immune features, and the prognostic significance of different SMARCA4 and SMARCA2 subunit deficiencies. Am J Surg Pathol 2022; 46 **:** 889–906. 34812766 10.1097/PAS.0000000000001836

[159] Horton RK , Ahadi M , Gill AJ , *et al*. SMARCA4/SMARCA2‐deficient carcinoma of the esophagus and gastroesophageal junction. Am J Surg Pathol 2021; 45 **:** 414–420. 33027072 10.1097/PAS.0000000000001599

[160] Schallenberg S , Bork J , Essakly A , *et al*. Loss of the SWI/SNF‐ATPase subunit members SMARCF1 (ARID1A), SMARCA2 (BRM), SMARCA4 (BRG1) and SMARCB1 (INI1) in oesophageal adenocarcinoma. BMC Cancer 2020; 20 **:** 12. 31906887 10.1186/s12885-019-6425-3 PMC6945480

[161] Neil AJ , Zhao L , Isidro RA , *et al*. SMARCA4 mutations in carcinomas of the esophagus, esophagogastric junction, and stomach. Mod Pathol 2023; 36 **:** 100183. 37054973 10.1016/j.modpat.2023.100183

[162] Le Loarer F , Watson S , Pierron G , *et al*. SMARCA4 inactivation defines a group of undifferentiated thoracic malignancies transcriptionally related to BAF‐deficient sarcomas. Nat Genet 2015; 47 **:** 1200–1205. 26343384 10.1038/ng.3399

[163] Rekhtman N , Montecalvo J , Chang JC , *et al*. SMARCA4‐deficient thoracic Sarcomatoid tumors represent primarily smoking‐related undifferentiated carcinomas rather than primary thoracic sarcomas. J Thorac Oncol 2020; 15 **:** 231–247. 31751681 10.1016/j.jtho.2019.10.023 PMC7556987

[164] Ahadi MS , Fuchs TL , Clarkson A , *et al*. Switch/sucrose‐non‐fermentable (SWI/SNF) complex (SMARCA4, SMARCA2, INI1/SMARCB1)‐deficient colorectal carcinomas are strongly associated with microsatellite instability: an incidence study in 4508 colorectal carcinomas. Histopathology 2022; 80 **:** 906–921. 34951482 10.1111/his.14612

[165] Morisue R , Kojima M , Suzuki T , *et al*. Common clinicopathological and immunological features of sarcomatoid carcinoma across organs: a histomorphology‐based cross‐organ study. Int J Cancer 2023; 153 **:** 1997–2010. 37548077 10.1002/ijc.34680

[166] Peng LS , Cui Q , Zhang C , *et al*. Neoadjuvant immunochemotherapy in Resectable NSCLC with SMARCA4 alterations. J Thorac Oncol 2026; 21 **:** 103506. 41161592 10.1016/j.jtho.2025.10.013

[167] Benson AB , Venook AP , Al‐Hawary MM , *et al*. Small bowel adenocarcinoma, version 1.2020, NCCN clinical practice guidelines in oncology. J Natl Compr Canc Netw 2019; 17 **:** 1109–1133. 31487687 10.6004/jnccn.2019.0043 PMC10191182

[168] Saigi M , Oliva M , Aliste L , *et al*. Clinical relevance of histologic subtypes in locally advanced esophageal carcinoma treated with pre‐operative chemoradiotherapy: experience of a monographic oncologic centre. PLoS One 2017; 12 **:** e0184737. 28931046 10.1371/journal.pone.0184737 PMC5607166

[169] Razenberg LG , van Gestel YR , Lemmens VE , *et al*. The prognostic relevance of histological subtype in patients with peritoneal metastases from colorectal cancer: a Nationwide population‐based study. Clin Colorectal Cancer 2015; 14 **:** e13–e19. 26140733 10.1016/j.clcc.2015.05.011

[170] Sentani K , Imai T , Kobayashi G , *et al*. Histological diversity and molecular characteristics in gastric cancer: relation of cancer stem cell‐related molecules and receptor tyrosine kinase molecules to mixed histological type and more histological patterns. Gastric Cancer 2021; 24 **:** 368–381. 33118117 10.1007/s10120-020-01133-w

[171] Arends MJ , Esposito I , Gill AJ , *et al*. Changes in the 6th edition of the World Health Organization classification of tumours of the digestive system. Histopathology 2026; 88 **:** 1295–1314. 41724188 10.1111/his.70116 PMC13128328

[172] Lizardo DY , Kuang C , Hao S , *et al*. Immunotherapy efficacy on mismatch repair‐deficient colorectal cancer: from bench to bedside. Biochim Biophys Acta Rev Cancer 2020; 1874 **:** 188447. 33035640 10.1016/j.bbcan.2020.188447 PMC7886024

